# Spatial and temporal autocorrelations affect Taylor's law for US county populations: Descriptive and predictive models

**DOI:** 10.1371/journal.pone.0245062

**Published:** 2021-01-07

**Authors:** Meng Xu, Joel E. Cohen

**Affiliations:** 1 Department of Mathematics, Pace University, New York, New York, United States of America; 2 Laboratory of Populations, The Rockefeller University and Columbia University, New York, New York, United States of America; 3 Earth Institute and Department of Statistics, Columbia University, New York, New York, United States of America; 4 Department of Statistics, University of Chicago, Chicago, Illinois, United States of America; University of Minnesota Duluth, UNITED STATES

## Abstract

Understanding the spatial and temporal distributions and fluctuations of living populations is a central goal in ecology and demography. A scaling pattern called Taylor's law has been used to quantify the distributions of populations. Taylor's law asserts a linear relationship between the logarithm of the mean and the logarithm of the variance of population size. Here, extending previous work, we use generalized least-squares models to describe three types of Taylor's law. These models incorporate the temporal and spatial autocorrelations in the mean-variance data. Moreover, we analyze three purely statistical models to predict the form and slope of Taylor's law. We apply these descriptive and predictive models of Taylor's law to the county population counts of the United States decennial censuses (1790–2010). We find that the temporal and spatial autocorrelations strongly affect estimates of the slope of Taylor's law, and generalized least-squares models that take account of these autocorrelations are often superior to ordinary least-squares models. Temporal and spatial autocorrelations combine with demographic factors (e.g., population growth and historical events) to influence Taylor's law for human population data. Our results show that the assumptions of a descriptive model must be carefully evaluated when it is used to estimate and interpret the slope of Taylor's law.

## Introduction

Understanding the spatial and temporal distribution and variation of species populations is fundamental in ecology and demography. Taylor's law (hereafter TL) states that the logarithm of the variance of population size (defined as count of individuals or count of individuals per unit area) is a linear function of the logarithm of the mean population size [1]. TL has been confirmed for thousands of biological taxa under various biological and environmental conditions [2], ranging from laboratory bacterial microcosms [3] to natural forest stands [4]; from agricultural crops [5,6] to wildlife animals [7]; and from free-living species to parasitized species [8,9]. TL has also been observed in non-biological systems [10–16].

In human demography, studies have tested and sometimes confirmed TL using sub-national (or national) human population size from Italy, Norway, United States, and the world [17–22]. Recent works have also tested and usually confirmed TL for age-specific annual human death rates in 12 countries [23,24]. Using the decennial census data, Xu and Cohen [22] found that US county population size obeys TL or its quadratic generalization, and compared the estimated TL slopes from different countries. They showed that significant temporal and spatial autocorrelation is present in the ordinary regression model residuals, which violated the assumptions of the ordinary regression models used for testing TL (i.e., a linear relationship between log(mean) and log(variance)). To our knowledge, no previous works on TL have used generalized regression models (which can incorporate the spatial and temporal structure) to describe TL for US county populations. On the other hand, no theoretical model (biological or statistical) has predicted the emergence of TL in US county populations. These gaps of knowledge impede the understanding of TL and limit its usefulness in demographic research.

Motivated by these gaps, here we extend the work by Xu and Cohen [22] to analyze the descriptive and predictive models of TL using US county population data. We have two goals. First, we will examine three types of TL (spatial hierarchical TL, spatial TL, and temporal TL) using generalized least-squares regressions with autocorrelated errors, and compare the results with those from the ordinary least-squares regressions. These comparisons will reveal the effect of temporal and spatial autocorrelation on the slope of TL, and facilitate establishing improved statistical standards for testing TL. Second, we will evaluate the assumptions and performance of three purely statistical models of TL using the US county population data. Such analyses will elucidate the mechanisms of spatial and temporal distributions of human population through the lens of TL.

## Materials and methods

### US census data and Taylor's law

County population count (number of individuals living in each county, historical or existing) in the United States was obtained from the decennial census from 1790 to 2010 [25]. Detailed descriptive statistics of the census data are given in Xu and Cohen [22]. As in [22], here "states" refers to states, territories, or equivalent primary administrative subdivisions of the United States, and "counties" refers to counties, parishes, or equivalent primary administrative subdivisions of any "state".

We study three types of Taylor's law (TL) and their corresponding quadratic generalizations (or quadratic Taylor's law, QTL). Specifically, in each census, we calculate a spatial mean and a spatial variance of county population counts across all counties within each state. If the logarithm of the spatial variance is well approximated by a linear function of the logarithm of the spatial mean across all states within a census, then the spatial hierarchical TL holds (approximately). If the same variables follow (approximately) a linear relationship across censuses within a state, then the spatial TL holds (approximately). Similarly, for each state, we calculate a temporal mean and a temporal variance of county population counts across all censuses within each county. If the logarithm of the temporal variance is (approximately) a linear function of the logarithm of the temporal mean across all counties within a state, then the temporal TL holds (approximately) for county populations within that state. If each set of logarithmic mean and logarithmic variance pairs follows a quadratic relationship, then the corresponding QTL holds. Xu and Cohen [22] tested these three types of TL using the ordinary least-squares (OLS) regression model with uncorrelated error *ε* (normally distributed, with zero mean and constant variance):

log(variance)=a+blog(mean)+ϵ. (Eq 1)

Here *a* and *b* are respectively the intercept and the slope of the corresponding TL. They also fitted the log(mean)-log(variance) relationship with an OLS quadratic regression model to examine each QTL:

$$log\;(variance)=c+d\;log\;(mean)+e{\left[log\;(mean)\right]}^{2}+\epsilon .$$ (Eq 2)

Here the quadratic coefficient *e* indicates the convexity (*e*>0) or concavity (*e*<0) in the relationship. Throughout log = log_10_.

### Descriptive models of Taylor's law

We reexamine the three types of TL by fitting generalized least-squares (GLS) regressions to the log(mean)-log(variance) data. This practice is warranted because the residuals of the OLS regressions had significant spatial and temporal autocorrelations (see S9-S14 Tables in [22]), which violated the assumption of uncorrelated errors [26]. These GLS regressions incorporate various autocorrelation error structures and may better describe the mean-variance relationships (Eqs 1 and 2). We compare the OLS and GLS regressions using the Akaike Information Criterion corrected for sample size (AICc) and evaluate the difference in TL slopes between the OLS and the best regression model (the one with the least AICc). For each set of log(mean) and log(variance) pairs, we calculate and compare the model AICc using the maximum likelihood method. We obtain the unbiased TL slope and standard error using the restricted maximum likelihood method [27]. Comparison of AICc shows the relative quality of the model based on the model likelihood and complexity. Some GLS models yield greater AICc than the OLS model because the GLS model contains more parameters in the correlation structure. It is possible that the increases in the number of parameters is greater than the increase of the model likelihood for some GLS models, increasing AICc. To evaluate the absolute goodness of fit of the model, we calculate the correlation coefficient between the observed variance and the predicted variance for each combination of TL and least-squares model.

For the spatial hierarchical TL across states in each census, we fit the corresponding log(spatial mean)-log(spatial variance) pairs using five spatially explicit GLS regression models with different spatial correlation structures separately: exponential, Gaussian, linear, rational quadratics, and spherical. These models describe the correlation of two mean-variance pairs as a decreasing function of the spatial distance between two states. For example, the Gaussian correlation structure models correlation as exp(-(*r*/*d*)^2^), where *d* is the range (the span of distances over which observations are correlated) and *r* is the distance between two observations. The mathematical forms of all correlation structures are listed in [27] (their Table 5.2). These correlation structures allow heteroscedastic errors (errors of unequal variance) in the regression model. For the spatial TL across censuses in each state, we fit the corresponding log(spatial mean)-log(spatial variance) pairs with three GLS regression models of different temporal correlation structures separately: order-1 autoregressive, order-1 moving average, and order-1 autoregressive order-1 moving average. These temporal correlation structures are decreasing functions of time lag and are described in [27]. For the temporal TL across counties in each state, we fit the corresponding log(temporal mean)-log(temporal variance) pairs with the same five spatially explicit GLS models of different spatial correlation structures separately, as we do for the spatial hierarchical TL.

For each county with changing geographic centroids between censuses (due to county boundary changes), we average the geographic coordinates (latitude and longitude) of the centroids across censuses to specify a single geographic location for that county. The total number of positive spatial mean-spatial variance pairs (across all censuses and states) is 958. The total number of positive temporal mean-temporal variance pairs (across all states and counties) is 3397. We fit each regression model to at least five finite log(mean)-log(variance) pairs across the corresponding scale, within a census or a state; if fewer pairs are available, we skip the analysis. No constraint is imposed on the number of units (i.e., counties within state, censuses for a county) used to generate one mean-variance pair. In a given census, the minimum number of counties in any state is two. In a given state, the minimum number of censuses for any county is two. Requiring at least 15 units per mean-variance pair does not change the overall finding of the descriptive models. The corresponding results based on at least 15 units per mean-variance pair are given in the supporting information (SI) S6 Appendix in S1 File.

For each individual regression model of TL, we derive the 95% interval estimate of TL's slope using the normal theory: point estimate ± *t*_0.025, n-1_×standard error. Here *t*_0.025, n-1_ is the critical *t* value with one-sided cutoff area of 0.025 and degree of freedom of n-1 (n being the number of finite log(mean)-log(variance) pairs) [26].

### Predictive models of Taylor's law

Besides the descriptive regression models for TL, we use three purely statistical models to predict TL's slope. For each predictive model and each type of TL, we evaluate the model assumptions using the US county data and compare the predicted slope with that obtained from the descriptive models. We summarize the model assumptions and fitting in the main text. We give details of assumption checking, prediction procedure, and software used in the SI.

#### a. Random samples of skewed distributions

Cohen and Xu [28] (hereafter CX) showed that random samples of independently and identically distributed (iid) observations of any skewed nonnegative distribution with finite third moment obey TL. CX gave explicit formulas to approximate the TL slope and its standard error (their eqns 3 and 5). Here, we first check the iid assumptions of the CX model against the US county data using various statistical hypothesis tests. We then predict TL slopes and their standard errors using the CX model for the county data.

#### b. Tweedie's exponential dispersion models

Kendal and Jørgensen [29,30] used Tweedie's exponential dispersion models to predict TL. Tweedie models are a family of three-parameter probability distributions and assume that observations follow the same distribution. We first evaluate the goodness of fit of the Tweedie models and other competing distribution models against the US county data. We then predict the point estimate and standard error of TL slope using the Tweedie models for the county data. Unlike the CX model that gives an explicit estimator of TL slope, Tweedie models estimate the TL slope numerically by fitting the empirical distribution using the maximum likelihood method.

#### c. Feasible sets of integer partitions

Any positive integer *N* can be partitioned into a sum of a given positive number *p* of nonnegative integers *N* = *n*_1_+⋯+*n_p_*, *n_i_*≥0, *i* = 1,…,*p*. In the theory of partitions, *p* is the number of "parts" and each summand *n_i_* is one part. For the spatial hierarchical TL and spatial TL, we identify the positive integer *N* with the population size (number of people) of a state and the parts with the population size of the counties in that state. For the temporal TL, we identify the positive integer *N* with the cumulative county population size (number of people) over all available censuses of a county and the parts with the county population size in one census.

For example, integer 2 (population of 2 people) can be partitioned into two parts (unordered parts: (2, 0) and (1, 1), or ordered parts: (2, 0), (1, 1), and (0, 2)). The resulting set of all possible partitions is called the feasible set. The partition (2, 0) has one zero part, while the partition (1, 1) has no zero parts. In our empirical application, a zero part would represent a county with no people (in the particular census). Throughout this work we used only unordered partitions including zero parts to predict TL, since there is no natural ordering of counties.

A feasible set can generate multiple variances at the same mean. (The example above yields variances of 2 = variance of {0, 2} and 0 = variance of {1, 1} for the same mean of 1.) Xiao, Locey, and White [31] (hereafter XLW) used feasible sets to predict TL slopes. Using the unordered integer partitions including zero parts, we predict TL using the XLW model for the US county data and we examine the similarity in distributions between the feasible sets and the empirical data. A concrete example of the application of unordered partitions to model hypothetical demographic data is given in the SI (S2 Appendix in S1 File, section C).

#### d. Model comparisons of TL's slopes

For each TL, we use the bootstrap to compare five estimates of TL's slope *b*: one from the ordinary least-squares linear regression (denoted as *b*_ols_), one from the best least-squares linear regression (denoted as *b*_reg_); one from the CX's formula (eqn 3 in [28]) (denoted as *b*_CX_); one from the Tweedie distribution model (denoted as *b*_T_); and one from the XLW model (including zero parts) (denoted as *b*_XLW_).

Specifically, for the spatial hierarchical TL and the spatial TL, we bootstrap 100 samples of county population counts from all counties within each combination of census and state. For the temporal TL, we bootstrap 100 samples of county population counties from all censuses within each combination of state and county. Each bootstrap sample contains the same number of counties (or censuses) as in the actual data (although the number of distinct counties (censuses) may differ due to sampling with replacement). For each bootstrap sample, we fitted regression and predictive models to estimate TL's slopes following the aforementioned methods. For the XLW model, we simulate 100 random partitions of state population count or historical county population count and generate 100 estimates of TL's slope for each bootstrap sample (following the method detailed in S2 Appendix in S1 File, section C). We use the median of the 100 estimated slopes as the point estimate of TL's slope for that bootstrap sample. For each census in the spatial hierarchical TL, and each state in the spatial TL and the temporal TL, the bootstrap samples yield 100 estimates of *b*_ols_, *b*_reg_, *b*_CX_, *b*_T_, and *b*_XLW_ separately. These procedures generate four sets of 100 sample differences between *b*_reg_ and each of the other four *b*'s respectively. We construct the 95% interval estimate of each slope difference using 2.5% and 97.5% percentiles of the corresponding set of sample differences. If a 95% interval estimate of a slope difference does not include zero, we infer that the best linear regression model gives an estimate of TL's slope that differs significantly from the slope estimate of another model.

We set the significance level of a hypothesis test at 0.05 and the confidence level at 95% for an interval estimate. Since hypothesis tests and model comparisons involve repeated tests for multiple spatial or temporal units, we use Bonferroni correction to offset the increased type-1 error (significance level = 0.05/number of tests (or comparisons)) caused by multiple comparisons. Results based on the Bonferroni correction are given in the SI. All data analysis and model fitting are performed in R [32].

## Results

### GLS regressions and descriptive model comparison

#### a. Spatial hierarchical TL

**Fig 1:**
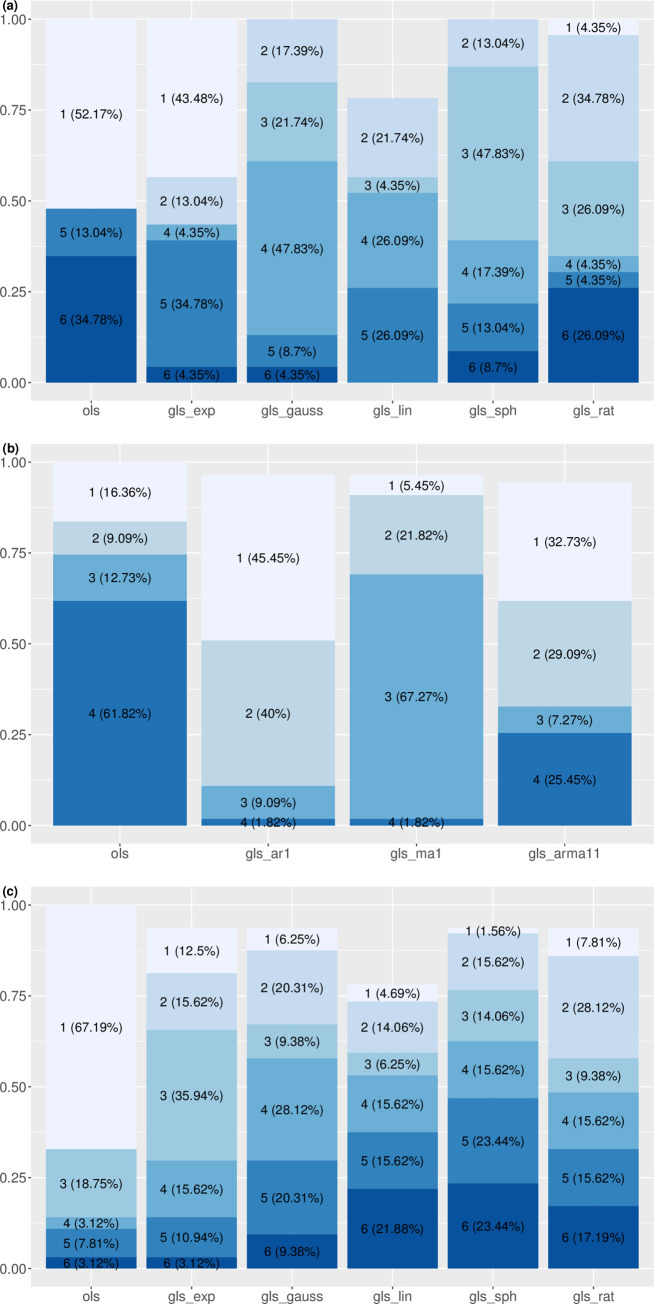
Rankings and proportions of least-squares linear regression models of (a) spatial hierarchical TL, (b) spatial TL, and (c) temporal TL. "ols" denotes the ordinary least-squares regression. "gls_exp", "gls_gauss","gls_lin","gls_sph", and "gls_rat" denote the generalized least-squares linear regression with the following spatial correlation structures respectively: exponential, Gaussian, linear, spherical, and rational quadratics. "gls_ar1", "gls_ma1", and "gls_arma11" denote the generalized least-squares linear regression with the following temporal correlation structures respectively: order-1 autoregressive, order-1 moving average, and order-1 autoregressive order-1 moving average. Number in front of each parenthesis represents the model rank according to AICc (model with the smallest AICc has rank 1 and model with the largest AICc has rank 6). Percentage in each parenthesis gives the proportion of censuses (23 for (a) spatial hierarchical TL) or states (55 for (b) spatial TL and 64 for (c) temporal TL) in which the model has the corresponding rank. For example, when fitting the spatial hierarchical TL, ordinary least-squares regression (ols) fit the mean-variance pairs more closely (by the AICc criterion) than the five other models in 52.17% of the 23 censuses (i.e., in 12 of 23 censuses). For some models the percentages do not add up to 100% because the corresponding maximum likelihood method fails to converge (for reasons not understood).

According to AICc, spatially correlated GLS linear regression is superior to the corresponding OLS linear regression (eqn 1) in describing the mean-variance relationship in most recent censuses (Tables 1 and S1). Of the 23 censuses, the best linear regression model (with the least AICc) is the OLS regression in 12 censuses (1790–1890, 1910), GLS regression with exponential spatial correlation in ten censuses (1900, 1920–2000), and GLS regression with rational quadratics spatial correlation in one census (2010) (Fig 1A). The best linear regressions yields significantly positive TL slopes in all censuses except 1790. The AICc weight of the best LS regression ranges from 0.22 to 0.58, with a median of 0.39. Correlation of the observed variance and the predicted variance of the best linear regression ranges from 0.52 to 0.92, with a median of 0.70 (S1 Fig, S2 Table). The OLS and the selected GLS differ slightly in their correlation (the correlation from the selected GLS is lower than that from OLS by 0.014–0.031 in 1900 and 1920–1970, and higher than that from OLS by 0.0029–0.027 in 1980–2010). Fitting to the bootstrap samples shows that TL's slope of the best linear regression model is significantly higher than that of the OLS regression in four censuses (1950, 1970, 1990, and 2000), and is not significantly different from that of the OLS regression in the remaining 19 censuses (S3 Table).

**Table 1:** Statistical models of TL and their summary statistics. The two descriptive models of TL are OLS (ordinary least-squares) and best LS (which is the OLS or GLS with the smallest AICc). The three predictive models of TL are the CX (Cohen-Xu) model, the Tweedie model, and the XLW (Xiao-Locey-White) models. 13 of the best LS models for the spatial TL and one of the best LS models for the temporal TL are GLS models that did not yield parameter estimates of TL due to failed convergence (for reasons not understood), resulting in respectively 42 (55–13) and 63 (64–1) best LS models. For each predictive model, the corresponding prediction of *b* is significantly less than (<) *b*_reg_ if the 95% interval estimate of the corresponding slope difference (from bootstrap samples) is less than zero, not significantly different from (≈) *b*_reg_ if the 95% interval estimate contains zero, and significantly greater than (>) *b*_reg_ if the 95% interval estimate is greater than zero.

model			TL		
type	name	statistics	spatial hierarchical	spatial	temporal
descriptive	OLS	no. of TL fitted	23	55	64
		no. of OLS selected	12	9	41
		range of *b*	1.54–2.47	0.21–5.54	1.49–3.93
		median of AICc weight	0.28	0	0.37
		median of correlation	0.70	0.97	0.97
	best LS	no. of TL fitted	23	42	63
		range of *b*	1.54–2.59	0.21–3.90	1.49–3.93
		median of AICc weight	0.39	0.76	0.44
		median of correlation	0.70	0.97	0.97
predictive	CX	range of *b*	3.21–7.80	1.09–5.56	0.63–5.56
		no. (%) of *b*_CX_ < *b*_reg_	0 (0)	9 (16)	13 (20)
		no. (%) of *b*_CX_ ≈ *b*_reg_	1 (4)	37 (67)	27 (42)
		no. (%) of *b*_CX_ > *b*_reg_	22 (96)	9 (16)	24 (38)
	Tweedie	range of *b*	1.97–2.87	1.29–3.08	1.65–3.02
		no. (%) of *b*_T_ < *b*_reg_	0 (0)	10 (18)	8 (13)
		no. (%) of *b*_T_ ≈ *b*_reg_	11 (48)	39 (71)	38 (59)
		no. (%) of *b*_T_ > *b*_reg_	12 (52)	6 (11)	18 (28)
	XLW	range of *b*	1.57–1.85	1.40–2.29	1.13–2.13
		no. (%) of *b*_XLW_ < *b*_reg_	10 (43)	32 (58)	45 (70)
		no. (%) of *b*_XLW_ ≈ *b*_reg_	13 (57)	23 (42)	19 (30)
		no. (%) of *b*_XLW_ > *b*_reg_	0 (0)	0 (0)	0 (0)

#### b. Spatial TL

Temporally correlated GLS linear regression models are superior to the OLS linear regression models in 46 of the 55 states according to AICc (Tables 1 and S4). Specifically, the best linear regression model selected by AICc is the GLS regression with a first-order autoregressive process in 25 states, GLS regression with a first-order moving average process in three states, and GLS regression with a first-order autoregressive moving average process in 18 states (Fig 1B). In 13 states where the GLS is the best model, the restricted maximum likelihood method fails to converge and does not yield the point estimate or standard error of TL slope. The best linear regression models yield significantly positive spatial TL slopes in all states except Alaska Territory, New Mexico Territory, and South Dakota. AICc weight of the best linear regression ranges from 0.022 to 1, with a median of 0.76. Correlation of the observed variance and the predicted variance from the best linear regression ranges from 0.44 to 1, with a median of 0.97 (S2 Fig, S5 Table). Difference in the correlation between the best GLS and the OLS is within a magnitude of 0.1 in all states except South Dakota, with a difference of -0.19. Interval estimate using bootstrap samples shows that TL's slopes do not differ between the best linear regression model and the OLS regression (S6 Table).

#### c. Temporal TL

For the temporal TL, the best linear regression model is OLS regression in 43 of the 64 states, GLS regression with exponential spatial correlation in eight states, GLS regression with Gaussian spatial correlation in four states, GLS regression with linear spatial correlation in three states, GLS regression with spherical spatial correlation in one state, and GLS regression with rational quadratics spatial correlation in five states (Tables 1 and S7, Fig 1C). In Washington, where the GLS regression with linear spatial correlation is the best model, the restricted maximum likelihood method fails to converge and does not yield the point estimate or standard error of TL slope. In all but three states (Alaska Territory, Arkansas Territory, and Wyoming Territory), the best linear regression model yields significantly positive TL slope. AICc weight of the best linear regression ranges from 0.23 to 1, with a median of 0.44. Correlation of the observed variance and the predicted variance from the best linear regression ranges from 0.066 to 1, with a median of 0.97 (S3 Fig, S8 Table). Difference of correlation between the selected GLS and the OLS is within a magnitude of 0.01 for all states except Maryland (with a magnitude of 0.044). Interval estimate using bootstrap samples shows that TL's slopes do not differ between the best linear regression model and the OLS regression (S9 Table).

### Assumptions of predictive models

The CX model assumes that samples of county population count are iid. The Tweedie model also assumes observations come from a single underlying distribution (which may be a compound distribution). The data are not consistent, in general, with these assumptions (S10–S20 Tables). Among the five distribution models that are used to describe the county population count, the Tweedie model and the Poisson lognormal model (see SI S2 Appendix in S1 File section B) yield AICc lower than that of the other three distributions in most censuses or states (S21–S23 Tables). Lastly, the XLW model yields random parts that are not significantly different from the empirical spatial distribution of county population count in 38.4% of the combinations of states and censuses (S24 Table), and not significantly different from the empirical temporal distribution in 51.9% of counties (S25 Table). Detailed results of the assumption checking are given in the SI.

### Comparisons of predictive models

#### a. Spatial hierarchical TL

**Fig 2:**
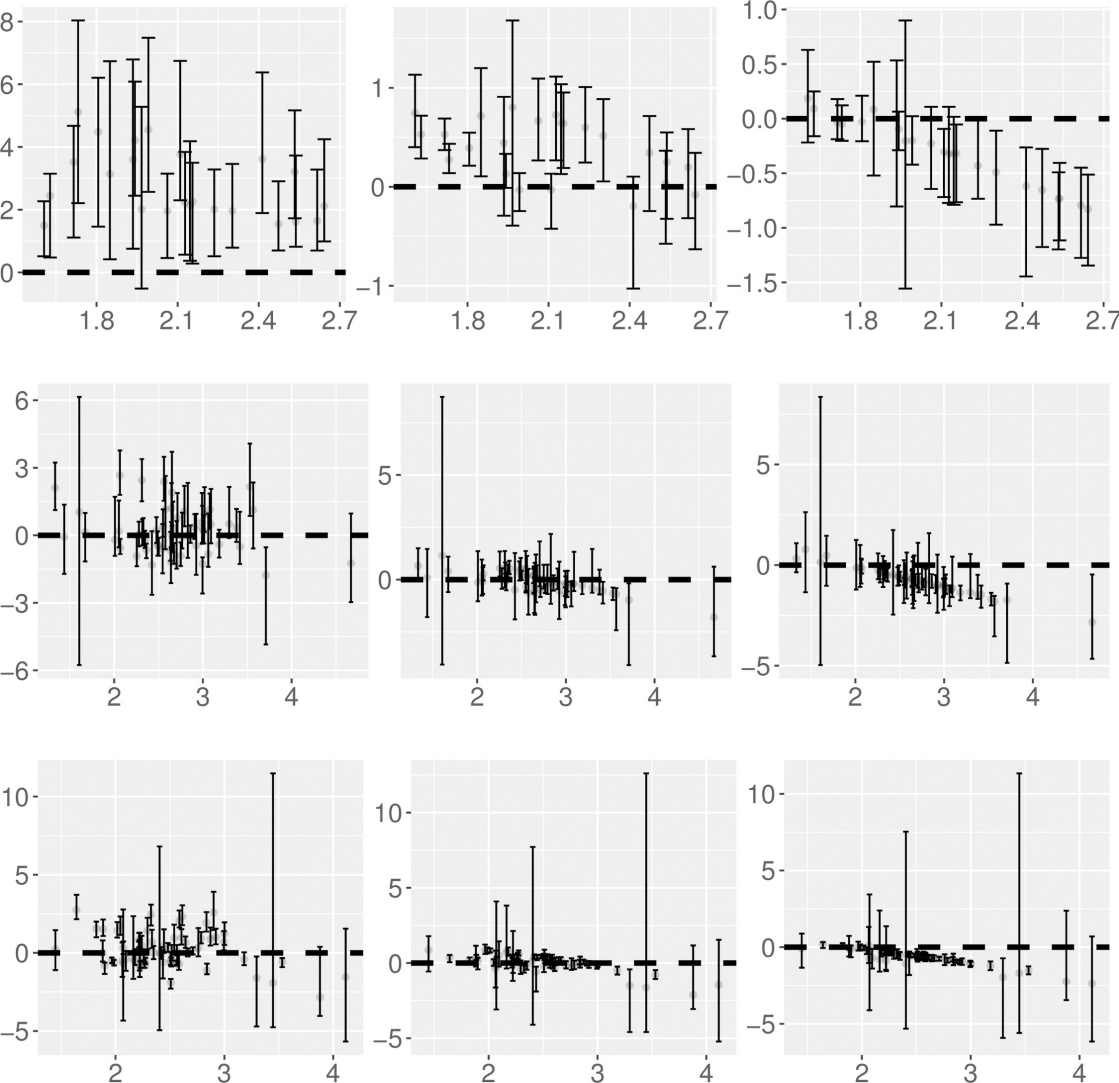
Difference of TL's slope between each predictive model and the best linear regression model against TL's slope estimate from the best linear regression model. The top, middle, and bottom rows correspond to spatial hierarchical TL, spatial TL, and temporal TL respectively. The left, middle, and right columns correspond to respectively the difference in slopes between the CX model and the best linear model, between the Tweedie model and the best linear model, and between the XLW model and the best linear model. Grey dots show the medians of the slope difference and error bars show the 95% interval estimates for each combination of TL, model, and census or state. The dashed horizonal line is the horizontal line at zero. An interval estimate above, across, or below the dashed line indicates that a model's slope estimate is significantly higher, not significantly different, or significantly lower than the corresponding slope estimate from the best linear model.

Using the bootstrap samples, the slope estimate of the spatial hierarchical TL predicted from the CX model (*b*_CX_) is significantly higher than that estimated from the best linear regression (*b*_reg_) for all 23 censuses except 1790 (Tables 1 and S3, Fig 2). The slope estimate predicted from the Tweedie model (*b*_T_) is not significantly different from *b*_reg_ in 11 censuses (1790, 1810, 1870–1950), and is significantly higher than *b*_reg_ in the other 12 censuses. The slope estimate from the XLW model (*b*_XLW_) is not significantly different from *b*_reg_ in 13 censuses (1790–1880, 1980, 1990, 2010), and is significantly lower than *b*_reg_ in the other ten censuses.

#### b. Spatial TL

Using the bootstrap samples, spatial TL's slopes predicted from the CX model (*b*_CX_) are not significantly different from those estimated from the best linear regression (*b*_reg_) in 37 of the 55 states (Tables 1 and S6, Fig 2). *b*_CX_ is significantly higher and lower than *b*_reg_ in nine and nine states respectively. Slope estimate from the Tweedie model (*b*_T_) does not differ significantly from *b*_reg_ in 39 states, and is significantly higher and significantly lower than *b*_reg_ in six and ten states respectively. For the XLW model, the predicted slope *b*_XLW_ is not significantly different from *b*_reg_ in 23 states, and is significantly lower than *b*_reg_ in 32 states.

#### c. Temporal TL

Using the bootstrap samples, temporal TL's slope estimated from the best linear regression (*b*_reg_) is not significantly different from *b*_CX_ in 27 of the 64 states. *b*_CX_ is significantly higher and significantly lower than *b*_reg_ in 24 and 13 states respectively (Tables 1 and S9, Fig 2). For the Tweedie model, *b*_T_ is not significantly different from *b*_reg_ in 38 states, and is significantly higher and significantly lower than *b*_reg_ in 18 and eight states respectively. For the XLW model, *b*_XLW_ is not significantly different from *b*_reg_ in 19 states, and is significantly lower than *b*_reg_ in the other 45 states.

## Discussion

### Implications of the descriptive models of TL

Several observations from the least-squares models of TL deserve interpretation.

First, for the spatial hierarchical TL, starting from the 1900 census (except the 1910 census), spatially correlated GLS models describe the mean-variance relationship of county population count better than the OLS regression (S1 Table). The assumption of uncorrelated errors in OLS is violated due to large spatial autocorrelation between states since 1900 (see S9 Table in [22]), which may be explained by the high population mobility contributed by several factors (e.g., employment, family, education attainment) [33,34]. For the censuses when GLS is the better linear regression model, the slope of TL estimated from GLS is higher (and significantly higher in four censuses) than that from OLS (S1 and S3 Tables). This suggests that OLS underestimates TL's slope when strong spatial autocorrelation is present in the data.

Second, for the spatial TL, GLS is better than OLS in describing the spatial mean-spatial variance relationship in most states (S4 Table), probably because the temporal autocorrelation of the spatial mean and spatial variance across censuses violates the assumption of the OLS model (see S11 Table in [22]). The slope estimates of TL yielded by the best GLS differ (but not significantly) from that by the OLS, and the difference depends on specific states (Tables 1 and S6). In other words, the influence of incorporating temporal autocorrelation in the regression models on TL's slope depends on the particular population growth trajectory of a state.

Third, for the temporal TL, OLS describes the temporal mean-temporal variance relationship across counties well in the majority of states (S7 Table). This result contrasts with the result from the spatial TL probably because the spatial autocorrelation among counties within a state is rather weak compared to the temporal autocorrelation among censuses within a state. Hence the assumption of spatially uncorrelated error for OLS (used to fit the temporal TL) is not violated in most states (see S13 Table in [22]). Similar to the spatial TL, the difference in the slopes of temporal TL estimated from the best linear regression and the OLS varies by state, and is not statistically significant in any state (Tables 1 and S9).

These results show that OLS models are not adequate to estimate the mean-variance relationship when spatial or temporal autocorrelations are present in the data. The GLS models that account for autocorrelation give different estimates of the TL slope from the OLS models. These differences depend on the specific type of TL, the population trend of the particular state, and the relative strength of the spatial and temporal autocorrelation. The most consistent pattern we observe is that OLS underestimates the slope of the spatial hierarchical TL when spatial autocorrelation is present (S1 Table). After 1900, the US experienced counter-urbanization that increased spatial autocorrelation across counties and states.

### Implications of the predictive models of TL

Assumption checking and model comparison of the predictive models of TL (the CX, Tweedie, and XLW models) further reveal the impact of temporal and spatial autocorrelation on TL.

Our statistical tests reject the null hypothesis that the county population counts are independently and identically distributed at various scales (S10–S20 Tables). The CX model yields significantly positive slopes of the spatial hierarchical TL, with values significantly higher than the empirical ones for most censuses (Tables 1 and S3). This observation implies that the CX model is not adequate to predict the slope of spatial hierarchical TL for at least some kinds of spatially correlated data at least when the homogeneity assumptions of the CX model are violated. For the spatial TL and the temporal TL, the CX model yields TL slopes that are significantly positive in all states (except Montana Territory and Wyoming Territory for the temporal TL) and similar to the empirical estimates in most states (Tables 1, S6 and S9), probably because in these two types of TL the effects of spatial and temporal autocorrelations are both present and may work in opposite directions, as we now describe.

While spatial autocorrelation may reduce TL's slope, temporal autocorrelation may increase TL's slope. When the data have both types of autocorrelations, the discrepancy in the slope between the best regression model and the CX model is dictated by the relative strength of the spatial and temporal autocorrelation in the specific state. Similar strength of the spatial and temporal autocorrelation may balance out the opposing effects on the slope of spatial TL and temporal TL. Our empirical analysis gives partial evidence supporting the claim in the previous paragraph. S2 Appendix in S1 File section A gives details, references, and source software for our methods.

For this analysis, we define spatial autocorrelation to be strong (or weak) if a state has at least (or less than) 25% of censuses with significant global Moran's I. We define temporal autocorrelation to be strong (or weak) if a state has at least (or less than) 80% of counties with significant temporal autocorrelation of lags of 1 through 4 by the Ljung-Box test. For the spatial TL, of the 31 states where *b*_CX_ is not significantly different from *b*_reg_ and the spatial and temporal autocorrelations are tested (S19, S20 and S24 Tables), we find that 15 of the 31 states show similar strength of spatial autocorrelation and temporal autocorrelation. For the temporal TL, of the 26 states where *b*_CX_ is not significantly different from *b*_reg_ and the spatial and temporal autocorrelations are tested (S19, S20 and S25 Tables), we find that 11 of the 26 states show similar strength of spatial autocorrelation and temporal autocorrelation. Examining the model predictions does not show that the discrepancy between the slope predicted by the CX model and *b*_reg_ is due to small sample sizes in a state.

For all types of TL, the Tweedie model yields significantly positive slopes that are similar to the corresponding empirical estimates in most censuses and states (Tables 1, S3, S6 and S9). Two aspects may explain this observation. First, even though the iid assumptions are rejected in most cases, the Tweedie model gives a reasonable description of the county population count distribution at most scales (comparable to the Poisson-lognormal model and better than other three models, see SI and S21–S23 Tables). A better fit to the observed count distribution may lead to better agreement between the Tweedie-model's predicted slope and the best regression estimate of the TL slope. Second, as a three-parameter distribution family, the Tweedie model is much more flexible than the CX model and the XLW models, which have no free parameters.

Lastly, for the spatial hierarchical TL, the XLW model yields TL's slopes that are significantly positive in all censuses and similar to the best linear regression estimate in all censuses except 1890–1970 and 2000 (Tables 1 and S3). The random partitions of state population count by the XLW model produce spatially correlated county population count predictions (since the sum of county population count equals the state population count), mimicking the spatial autocorrelation in the data and producing estimates of TL slope consistent with those of the best least-squares regression. During 1890–1970, the great migration of farming and African-American populations to urban areas increased the spatial aggregation (or reduced the spatial autocorrelation) of county populations [35,36], while the XLW model fails to capture such change in the spatial autocorrelation and yields smaller TL slopes. This finding corroborates our results on the descriptive models that spatial autocorrelation can reduce the slope of TL.

For the spatial TL, the slope predicted by the XLW model is significantly positive in most states and significantly smaller than the best linear regression estimated slope in 32 states (Tables 1 and S6), likely because the XLW model fails to capture the strong temporal autocorrelation across censuses for those states. For the temporal TL, the slope predicted by the XLW model is significantly positive in all but one state (Montana Territory), and significantly smaller than the best linear regression estimate in 45 states (Tables 1 and S9), probably because the temporal autocorrelation embedded in the random partitions of total historical county population count is not as strong as that in the original data. Based on these observations, we speculate that temporal autocorrelation can increase the slopes of the spatial TL and the temporal TL.

### Conclusions

Our descriptive models of TL show that, when temporal and spatial autocorrelations in the data violate the assumption of the OLS models, GLS provides a better description of the mean-variance relationship and yields different TL slope estimates. This finding suggests that, in future testing of TL, the autocorrelation structure of the data should be checked when fitting regression models and comparing parameter estimates.

TL's slope mostly falls between one and two for non-human populations [31,37]. In the current work, for all three types of TL, we find that the best linear regression model yields TL's slope that is significantly higher than two in some censuses or states (seven of 23 censuses for the spatial hierarchical TL, 12 of 55 states for the spatial TL, and 34 of 64 states for the temporal TL, see S1, S4 and S7 Tables). This indicates that human populations exhibit different spatial and temporal aggregation patterns than non-human populations. In addition to the resources and environments that constrain the growth and movement of non-human populations, the spatiotemporal dynamics of human populations are also influenced by political, economic, and cultural conditions. These variables may have accelerated the spatial and temporal aggregation and resulted in enlarged TL's slope for human populations in our US census data, but we do not claim to have identified a specific mechanism for the difference in slopes between most non-human and these human populations.

All three predictive models yield significantly positive slopes of TL, regardless of the types of TL and regardless of whether the model assumptions are satisfied (Table 1). This means that the power-law form of TL can be generated robustly by multiple statistical mechanisms, including random sampling of skewed distribution, probability distribution models, and random partitions of integers (see also [38]). However, TL slopes predicted by these models are not always consistent with the slopes estimated by the best regression models. In addition to spatial and temporal autocorrelations, the estimated slope of TL also depends on the population growth patterns in various states and historical demographic events and trends.

Recent works used statistical theory to study the relationship between spatial or temporal autocorrelations and TL for ecological populations [39,40]. For example, Reuman et al. [40] showed analytically that spatial synchrony (a special form of spatial autocorrelation to depict the spatial covariation in population time series, see [41]) decreases the slope of spatial TL. Their finding qualitatively predicts our empirical results. It would be interesting to test whether their formula (see eqn 2 in [40]) can yield better quantitative agreement to the best-regression TL's slope than the CX model for the US county population data. On the other hand, Cohen and Saitoh [39] used a modified Gompertz model (a density-dependent time series model with temporal autocorrelation), which, when combined with spatial correlation (synchrony), successfully predicted the OLS slopes of the temporal TL and the spatial TL for rodent populations. Whether a mathematical model can adequately describe the population dynamics and the corresponding TL's slope for human population data remains to be seen.

We have shown that spatial and temporal autocorrelations can systematically affect TL slopes. Our empirical analysis of TL in the US county population counts shows that intensified spatial autocorrelation can decrease the slope and intensified temporal autocorrelation can increase the slope. Using statistical models with violated assumptions can lead to erroneous estimation and interpretation of TL's slope. Future works should investigate the effect of spatial and temporal autocorrelations on TL at other spatial scales (e.g., division of state into census tracts or townships). They should also study other countries and other statistical models that can incorporate population growth patterns to estimate the parameters and evaluate the goodness of fit of TL.

## Supporting information

S1 Fig Predicted spatial variance of county population count against observed spatial variance for each census. Each marker shows one observed variance-predicted variance pair within a state in one census. Different markers denote the variance predicted from the ordinary least-squares regression (Δ) or the best least-squares regression with the smallest AICc (○). Predictions from the best least-squares regression are missing in some censuses because the ordinary least-squares is the best model. Dashed lines are the one-to-one reference lines.
(PDF)

S2 Fig Predicted spatial variance of county population count against observed spatial variance for each state. Each marker shows one observed variance-predicted variance pair in one census within a state. Markers and lines are defined in S1 Fig. Predictions from the best least-squares regression are missing in some censuses because either the ordinary least-squares is the best model or the generalized least-squares regression fails to yield any prediction.
(PDF)

S3 Fig Predicted temporal variance of county population count against observed temporal variance for each state. Each marker shows one observed variance-predicted variance pair in one county within a state. Markers and lines are defined in S1 Fig. Predictions from the best least-squares regression are missing in some censuses because either the ordinary least-squares is the best model or the generalized least-squares regression fails to yield any prediction.
(PDF)

S1 Table Descriptive model summary of the spatial hierarchical TL for each census (year). n is the number of finite log(mean)-log(variance) pairs (here, the number of states) in each fitting. OLS is the ordinary least-squares linear regression and best LS is the least-squares linear regression with the smallest AICc. mod shows the name of the best LS. gls_exp and gls_rat are respectively the generalized least-squares linear regression with exponential spatial correlation and rational quadratics spatial correlation. est and std err are respectively the point estimate and standard error of the estimate. lower and upper are respectively the lower and upper bounds of the slope estimates with 95% confidence level. AICc and AICc wt are respectively the Akaike Information Criterion corrected for the number of parameters and the AICc weight of the corresponding model among all fitted models in a census. A model with the smaller AICc (or greater AICc wt) is better. cor is the correlation coefficient of the observed variance and the predicted variance from each model. ΔAICc is AICc of the best LS minus the AICc of the OLS.
(XLSX)

S2 Table Correlation coefficient (r) between the observed variance and predicted variance using the least-squares regressions, for the spatial hierarchical TL tested in each census (year). mod lists gls_exp, gls_gauss, gls_lin, gls_rat, and gls_sph, which are respectively the generalized least-squares regressions with exponential, Gaussian, linear, rational quadratics, and spherical spatial correlation. ols is the ordinary least-squares regression.
(XLSX)

S3 Table Interval estimate of the slope difference of spatial hierarchical TL between the best linear model (breg) and the ordinary least-squares regression (bols), Cohen-Xu model (bCX), Tweedie model (bT), and Xiao-Locey-White model (bXLW) in each census (Year). Each interval estimate is derived from 100 estimated slope differences using 100 bootstrap samples of the county population data. Lower and upper values are respectively the 2.5% and 97.5% percentiles of the 100 slope differences.
(XLSX)

S4 Table Descriptive model summary of the spatial TL for each state. gls_ar1, gls_ma1, and gls_arma11 in the best LS are respectively the generalized least-squares linear regressions with first-order autoregressive component, first-order moving average component, and first-order autoregressive and first-order moving average components. n is the number of finite log(mean)-log(variance) pairs (here, the number of censuses) in each fitting. Other notations are defined as in S1 Table.
(XLSX)

S5 Table Correlation coefficient (r) between the observed variance and predicted variance using the least-squares regressions, for the spatial TL tested in each state. gls_ar1, gls_ma1, and gls_arma11 are respectively the generalized least-squares regressions with first-order autoregressive component, first-order moving-average component, and first-order autoregression and first-order moving-average component. ols and r are defined in S2 Table.
(XLSX)

S6 Table Interval estimate of the slope difference of spatial TL between the best linear model (breg) and the ordinary least-squares regression (bols), Cohen-Xu model (bCX), Tweedie model (bT), and Xiao-Locey-White model (bXLW) in each state (State). S3 Table gives the method for interval estimation.
(XLSX)

S7 Table Descriptive model summary of the temporal TL for each state. gls_gauss, gls_sph, and gls_lin are respectively the generalized least-squares with Gaussian spatial correlation, spherical spatial correlation, and linear spatial correlation. n is the number of finite log(mean)-log(variance) pairs (here, the number of counties) in each fitting. Other notations are defined as in S1 Table.
(XLSX)

S8 Table Correlation coefficient (r) between the observed variance and predicted variance using the least-squares regressions, for the temporal TL tested in each state. mod and r are defined in S2 Table.
(XLSX)

S9 Table Interval estimate of the slope difference of temporal TL between the best linear model (breg) and the ordinary least-squares regression (bols), Cohen-Xu model (bCX), Tweedie model (bT), and Xiao-Locey-White model (bXLW) in each state (State). S3 Table gives the method for interval estimation.
(XLSX)

S10 Table Results of one-way analysis of variance of the state-specific spatial means of county population counts among states within each census (year). df is the degrees of freedom. sumsq is the sum of squares, meansq is the mean sum of squares, statistic is the F statistic, and p_value is the p value for the corresponding test.
(XLSX)

S11 Table Results of one-way analysis of variance of the spatial means of county population counts across censuses within each state. p_value is defined in S10 Table.
(XLSX)

S12 Table Results of one-way analysis of variance of the temporal means of county population counts across counties within each state. p_value is defined in S10 Table.
(XLSX)

S13 Table Bartlett test of homogeneity of the spatial variances of county population counts across states within each census (year). statistic is the chi-square test statistic. p_value is defined in S10 Table.
(XLSX)

S14 Table Bartlett test of homogeneity of the spatial variances of county population counts across censuses within each state. p_value is defined in S10 Table.
(XLSX)

S15 Table Bartlett test of homogeneity of the temporal variances of county population counts across counties within each state. p_value is defined in S10 Table.
(XLSX)

S16 Table Anderson-Darling test of identical spatial distribution of county population counts across states within each census. p_value is defined in S10 Table.
(XLSX)

S17 Table Anderson-Darling test of identical spatial distribution of county population counts across censuses within each state. p_value is defined in S10 Table.
(XLSX)

S18 Table Anderson-Darling test of identical temporal distribution of county population counts across counties within each state. p_value is defined in S10 Table.
(XLSX)

S19 Table Summary statistics of global Moran's I for spatial autocorrelation of county population counts within each combination of state and year. moran_exp and moran_obs are respectively the expected Moran's I and the observed Moran's I. p_ran and z_ran are respectively the p-value and the z score calculated from the randomization null hypothesis test (with undefined values denoted by NA). num_county is the number of counties.
(XLSX)

S20 Table Summary statistics of the Ljung-Box test of temporal autocorrelation (lag 1 to lag 5) of historical county population counts over years, for each combination of state and county. num_census is the number of censuses. tcor_lagX (X = 1,2,3,4,5) is the p-value of the test with a lag of 10X years.
(XLSX)

S21 Table Summary statistics of five distribution models of county population counts within each census (year). size is the number of counties. lik_max is the approximate maximum likelihood of the Tweedie model. aicc_xxx gives the AICc of the Tweedie model (xxx = tweedie) and of each of the other four distributions models: log series distribution (xxx = logseries), negative binomial distribution (xxx = negbinom), Poisson-lognormal distribution (xxx = poilog), and Zipf distribution (xxx = zipf). wt_xxx gives the AICc weight of each of the five distribution models.
(XLSX)

S22 Table Summary statistics of five distribution models of county population counts within each state (within at least five censuses in each state). size is the number of combinations of censuses and counties. Other columns are defined in S21 Table.
(XLSX)

S23 Table Summary statistics of five distribution models of county population counts within each state (within at least five counties in each state). size is the number of combinations of censuses and counties. Other columns are defined in S21 Table.
(XLSX)

S24 Table p-value of the 2-sample Kolmogorov-Smirnov (KS) test of the null hypothesis that the observed county population count distribution is identical to the predicted county population county distribution from the XLW model (including zero), for each combination of state and year. ks_p is the p-value of the KS test.
(XLSX)

S25 Table p-value of the 2-sample Kolmogorov-Smirnov (KS) test of the null hypothesis that the observed historical county population count distribution is identical to the predicted historical county population county distribution from the XLW model (including zero), for every county. ks_p is the p-value of the KS test.
(XLSX)

S26 Table Descriptive model summary of the spatial hierarchical quadratic TL for each census (year). OLS is the ordinary least-squares quadratic regression and best LS is the least-squares quadratic regression with the smallest AICc. n is the number of finite log(mean)-log(variance) pairs (here, the number of states) in each fitting. Other notations are defined in S1 Table.
(XLSX)

S27 Table Descriptive model summary of the spatial quadratic TL for each state. OLS is the ordinary least-squares quadratic regression and best LS is the least-squares quadratic regression with the smallest AICc. n is the number of finite log(mean)-log(variance) pairs (here, the number of censuses) in each fitting. Other notations are defined in S1 and S4 Tables.
(XLSX)

S28 Table Descriptive model summary of the temporal quadratic TL for each state. OLS is the ordinary least-squares quadratic regression and best LS is the least-squares quadratic regression with the smallest AICc. n is the number of finite log(mean)-log(variance) pairs (here, the number of counties) in each fitting. Other notations are defined in S1 and S7 Tables.
(XLSX)

S29 Table Proportion of states that show significant spatial autocorrelation of county population counts within each census, using five different ways of computing the Moran's I. Xneighbor_Y (X = 4 or 8, Y = binary or bisquare) is the binary (weight = 1 for distances less than or equal to the distance of the furthest neighbour, 0 otherwise) or bi-square (weight = (1-(distance of neighbor/distance of the furthest neighbor)^2)^2 or 0 otherwise) weighting scheme with 4 neighbors or 8 neighbors. allcounty_bisquare uses the bi-square weighting scheme for all counties in a state.
(XLSX)

S30 Table Interval estimates of the spatial hierarchical TL slope (b) using bootstrap samples for each census (year), using the CX model (cx) and the ordinary least-squares (ols) regression separately. b and b_se are respectively the point estimate and the standard error of b obtained from the census data. b_boot_withinyear_lower (or upper) gives respectively the 95% lower (or upper) bound of b estimated from 500 samples bootstrapped within each year. b_boot_withinyearstate_lower (or upper) gives respectively the 95% lower (or upper) bound of b estimated from 500 samples bootstrapped within each combination of year and state.
(XLSX)

S31 Table Interval estimates of the spatial TL slope (b) using bootstrap samples for each state, using the CX model (cx) and the ordinary least-squares (ols) regression separately. b and b_se are defined in S30 Table. b_boot_withinstate_lower (or upper) gives respectively the 95% lower (or upper) bound of b estimated from 500 samples bootstrapped within each state. b_boot_withinstateyear_lower (or upper) gives respectively the 95% lower (or upper) bound of b estimated from 500 samples bootstrapped within each combination of state and year.
(XLSX)

S32 Table Interval estimates of the temporal TL slope (b) using bootstrap samples for each state, using the CX model (cx) and the ordinary least-squares (ols) regression separately. b and b_se are defined in S30 Table. b_boot_withinstate_lower (or upper) gives respectively the 95% lower (or upper) bound of b estimated from 500 samples bootstrapped within each state. b_boot_withinstatecounty_lower (or upper) gives respectively the 95% lower (or upper) bound of b estimated from 500 samples bootstrapped within each combination of state and county.
(XLSX)

S33 Table Summary statistics of the best regression models (mod_best) of the spatial hierarchical TL for each census (year). n is the number of finite log(mean)-log(variance) pairs (here, the number of states) in each fitting. Each mean-variance pair is generated using at least 15 county population counts within a state. Model names are defined in S2 Table. "est_slp" and "err_slp" are respectively the slope estimate and slope standard error. "aicc" is the model AICc.
(XLSX)

S34 Table Summary statistics of the best regression models (mod_best) of the spatial TL for each state. n is the number of finite log(mean)-log(variance) pairs (here, the number of censuses) in each fitting. Each mean-variance pair is generated using at least 15 county population counts within a state. Model names are defined in S4 Table. "est_slp", "err_slp" and "aicc" are defined in S33 Table.
(XLSX)

S35 Table Summary statistics of the best regression models (mod_best) of the temporal TL for each state. n is the number of finite log(mean)-log(variance) pairs (here, the number of counties) in each fitting. Each mean-variance pair is generated using at least 15 historical population counts of a county. Model names are defined in S2 Table. "est_slp", "err_slp" and "aicc" are defined in S33 Table.
(XLSX)

S36 Table Spatial hierarchical TL's slope across five models in each census (year). The models are cx (Cohen-Xu model), reg_ols (ordinary least-squares linear regression), reg_best (least-squares linear regression with the smallest AICc), tweedie (Tweedie model), and xlw (Xiao-Locey-White model). n is the number of finite mean-variance pairs used in each model fitting. The last four columns show TL slope (b), standard error of the slope (b_se), and lower bound of the slope (b_lower) and upper bound of the slope (b_upper) using adjusted critical t value (upper tail probability is 0.025/23) according to the Bonferroni correction.
(XLSX)

S37 Table Spatial TL's slope across five models in each state. Notations are defined as in S36 Table. Lower and upper bounds of the slope are derived using adjusted critical t (upper tail probability is 0.025/55).
(XLSX)

S38 Table Temporal TL's slope across five models in each state. Notations are defined as in S36 Table. Lower and upper bounds of the slope are derived using adjusted critical t (upper tail probability is 0.025/64).
(XLSX)

S1 File (DOCX)
