# Supplementary figures and images for: Spatial and temporal autocorrelations affect Taylor's law for US county populations: Descriptive and predictive models
Source: PLoS One. 2021 Jan 7;16(1):e0245062. doi: 10.1371/journal.pone.0245062 (PMC7790542; doi:10.1371/journal.pone.0245062)

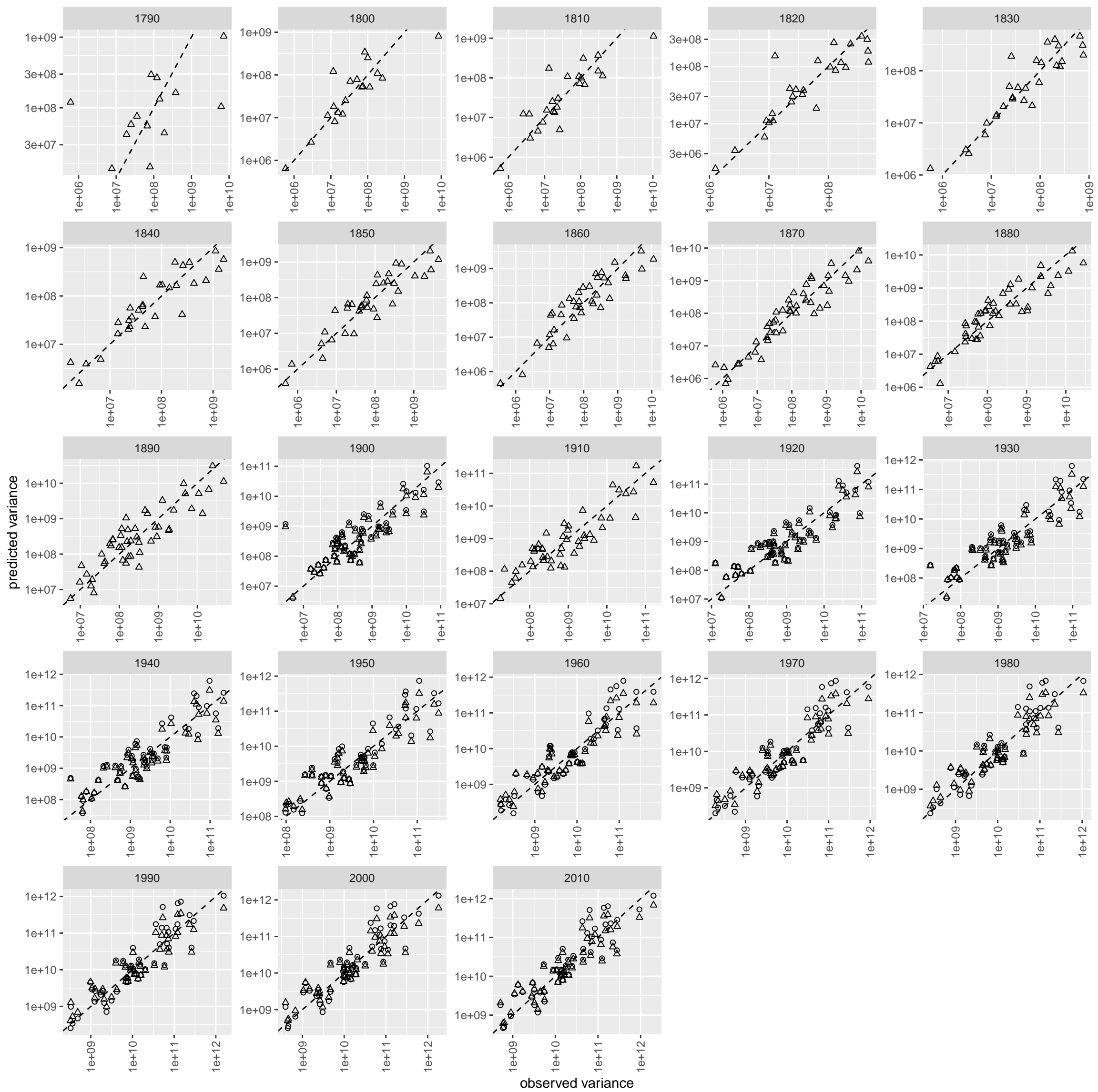

Supplement: S1 Fig — Each marker shows one observed variance-predicted variance pair within a state in one census. Different markers denote the variance predicted from the ordinary least-squares regression (Δ) or the best least-squares regression with the smallest AICc (○). Predictions from the best least-squares regression are missing in some censuses because the ordinary least-squares is the best model. Dashed lines are the one-to-one reference lines. (PDF) [file pone.0245062.s001.pdf]

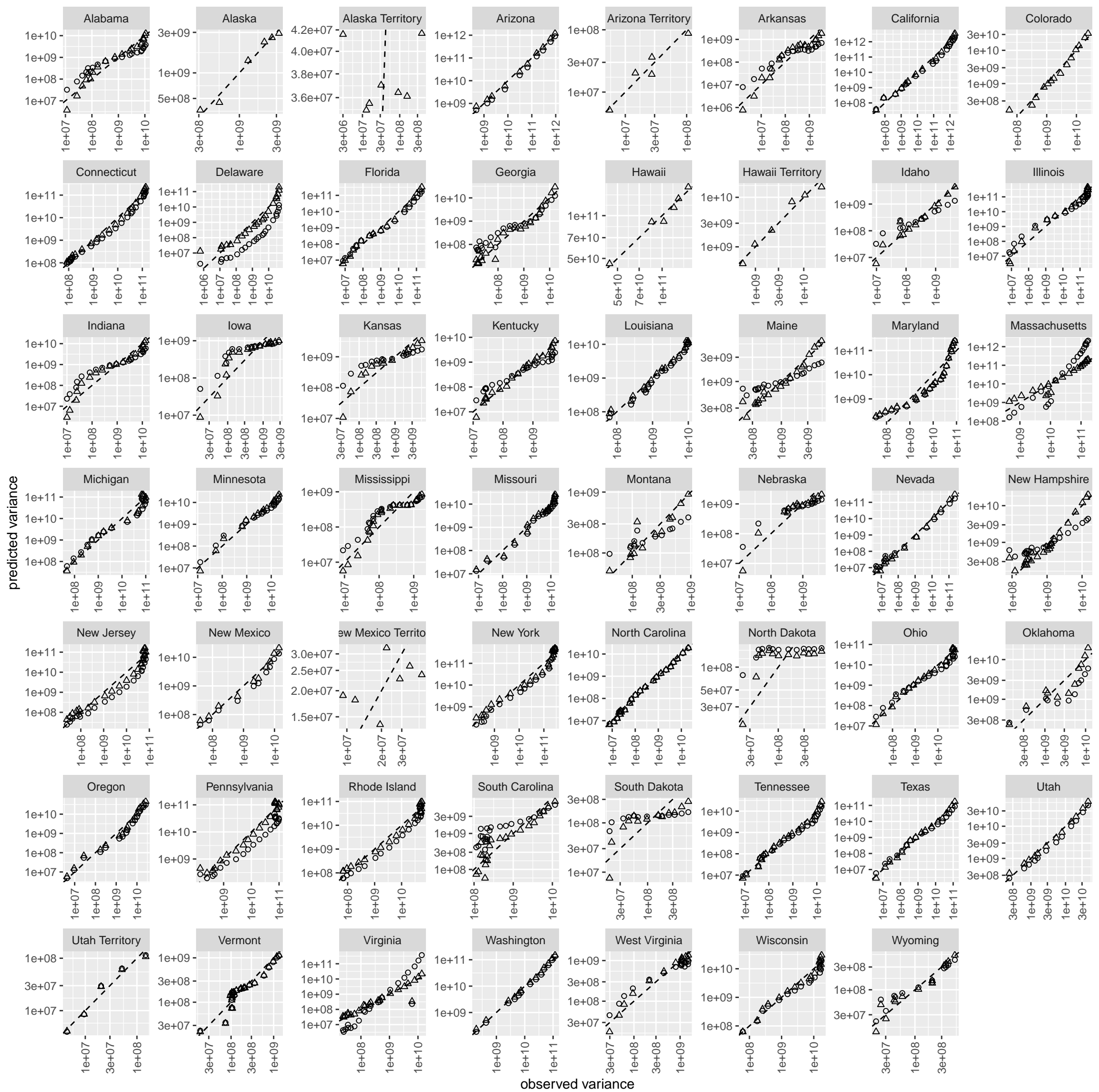

Supplement: S2 Fig — Each marker shows one observed variance-predicted variance pair in one census within a state. Markers and lines are defined in S1 Fig. Predictions from the best least-squares regression are missing in some censuses because either the ordinary least-squares is the best model or the generalized least-squares regression fails to yield any prediction. (PDF) [file pone.0245062.s002.pdf]

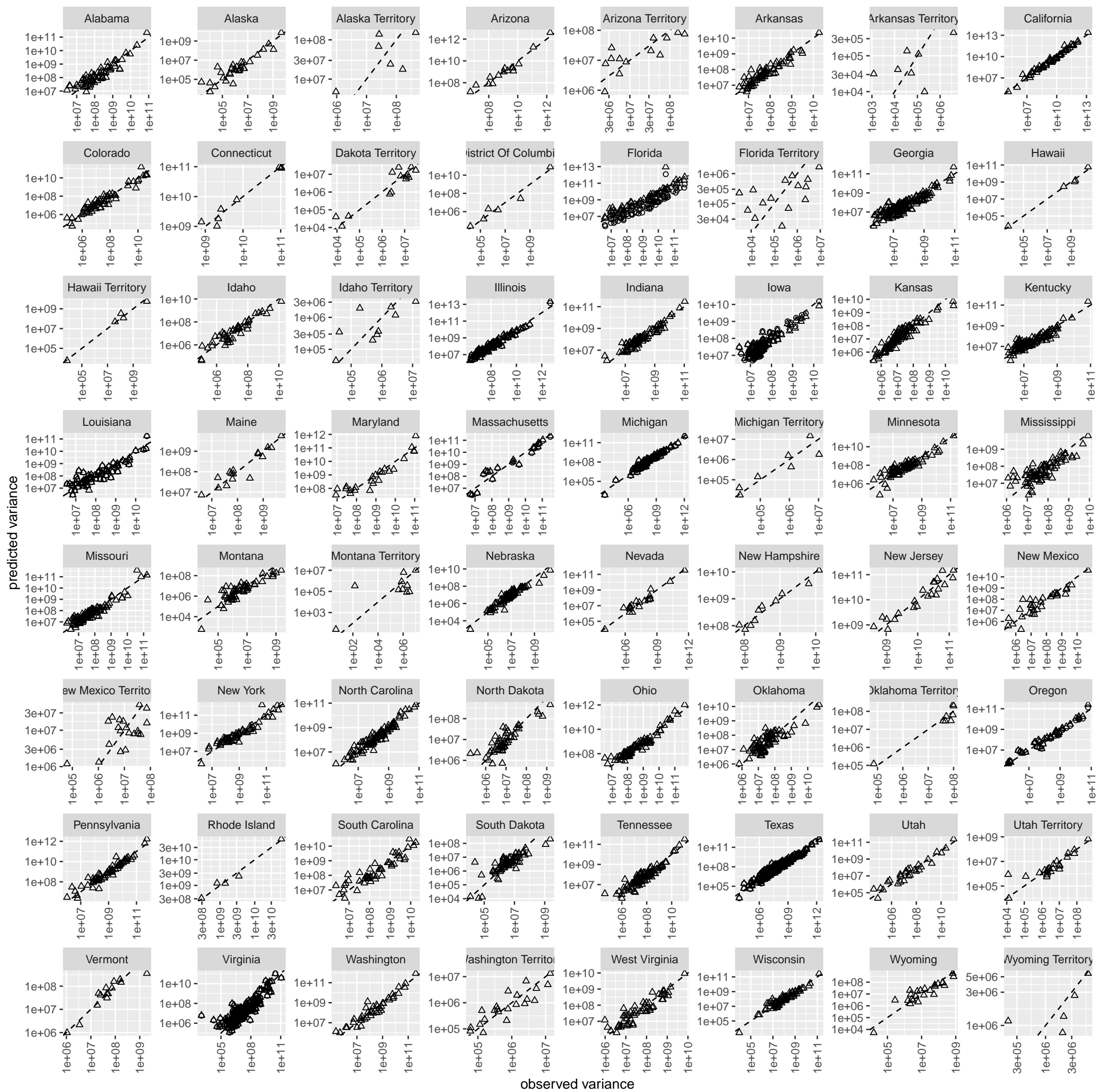

Supplement: S3 Fig — Each marker shows one observed variance-predicted variance pair in one county within a state. Markers and lines are defined in S1 Fig. Predictions from the best least-squares regression are missing in some censuses because either the ordinary least-squares is the best model or the generalized least-squares regression fails to yield any prediction. (PDF) [file pone.0245062.s003.pdf]
