# Supplementary material for: Spatial and temporal autocorrelations affect Taylor's law for US county populations: Descriptive and predictive models
Source: PLoS One. 2021 Jan 7;16(1):e0245062. doi: 10.1371/journal.pone.0245062 (PMC7790542; doi:10.1371/journal.pone.0245062)
Supplement: S1 File — (DOCX) [file pone.0245062.s042.docx]

Spatial and temporal autocorrelations affect Taylor's law for US county populations: descriptive and predictive models

Short title: space-time autocorrelation and Taylor's law

Meng Xu^1^ and Joel E. Cohen^2,3,4,*^

^1^Department of Mathematics, Pace University, New York, New York, United States of America

^2^Laboratory of Populations, The Rockefeller University and Columbia University, New York, New York, United States of America

^3^Earth Institute and Department of Statistics, Columbia University, New York, New York, United States of America

^4^Department of Statistics, University of Chicago, Chicago, Illinois, United States of America

^*^Corresponding author

E-mail: [*cohen@rockefeller.edu*](mailto:cohen@mail.rockefeller.edu)

# SUPPORTING INFORMATION

# Appendix S1: Results on the descriptive least-squares models of the quadratic TL

### A. Spatial hierarchical quadratic TL

The best quadratic model is OLS regression (eqn 2) in 12 censuses (1800-1890, 1910), GLS regression with exponential spatial correlation in five censuses (1900, 1920-1950), and GLS regression with rational quadratics spatial correlation in six censuses (1960-2010) (Table S26). Quadratic coefficients of the best quadratic regression models are not significantly different from zero in 17 censuses (1790-1950) and significantly negative in six censuses (1960-2010). AICc of the best linear model is smaller (or larger) than that of the best quadratic model in 16 (or 7) censuses. Correlation of the observed variance and the predicted variance differs slightly between the best linear model and the best quadratic model. The magnitude of the difference ranges from 0.0004 to 0.066.

### B. Spatial quadratic TL

GLS regression with a first-order autoregressive process is the best quadratic model in 19 states (Table S27). GLS regression with a first-order moving average process is the best quadratic model in nine states. GLS regression with a first-order autoregressive moving average process is the best quadratic model in 11 states. In two states (Arizona Territory and Utah Territory), the AICc of the OLS regression is undefined due to the limited number of censuses (five in each state). In seven states the restricted maximum likelihood method fails to converge in the best quadratic regression model and does not yield the point estimate or standard error of quadratic coefficient. Among the best quadratic models, eight states have a significantly negative quadratic coefficient and four states have a significantly positive quadratic coefficient. AICc of the best linear model is smaller (or larger) than that of the best quadratic model in 24 (or 31) states. Correlation of the observed variance and the predicted variance differs between the best linear regression and the best quadratic regression within a magnitude of 0.1 in all but three states. The difference of correlation between the best quadratic model and the best linear model is -0.54 in Alaska Territory, 0.13 in New Jersey, and 0.23 in South Dakota.

### C. Temporal quadratic TL

The best quadratic model is OLS regression in 41 states, GLS regression with exponential spatial correlation in 13 states, GLS regression with Gaussian spatial correlation in four states, GLS regression with linear spatial correlation in two states, GLS regression with spherical spatial correlation in one state, and GLS regression with rational quadratics spatial correlation in three states (Table S28). For Hawaii and Hawaii Territory, GLS regression is not fitted due to the lack of county coordinates, and OLS regression yields undefined AICc due to small sample sizes. Among the best quadratic models, the quadratic coefficient is significantly positive in four states (Iowa, Minnesota, Nebraska, and South Dakota) and significantly negative in six states (Florida, Illinois, Indiana, Maryland, Missouri, and Ohio). AICc of the best linear model is smaller (or larger) than that of the best quadratic model in 35 (or 27) states. Correlation of the observed variance and the predicted variance differs between the best quadratic model and the best linear model within a magnitude of 0.2 for all but three states. The difference in correlation equals -0.46 in Alaska Territory, 0.28 in Maryland, and 0.51 in Florida.

# Appendix S2: Statistical details of the predictive models of TL

### A. Random samples of skewed distributions

For independently and identically distributed (iid) samples, Cohen and Xu (2015) (hereafter CX) showed that the slope of TL is approximated by the ratio of the skewness over the coefficient of variation of the distribution when the sample size and number of samples are large. To estimate the standard error of the estimated slope requires a finite fourth moment. The CX model has no free parameters and involves no curve-fitting. This model provides a "null" model of TL, that is, a model devoid of mechanism other than random variation from a single underlying (positively skewed) distribution. Deviations from this model require some scientific or statistical explanation, but agreement with this model requires no assumption beyond simple random sampling. Reuman et al. (2017, their Supplement S2) generalized the CX model to spatially correlated and temporally independent observations.

We test the iid assumption of the CX model for the three types of TL using US county population count. Specifically, we test the equality of means and variances of county population count using the analysis of variance and the Bartlett test respectively, at various scales. Within each census, we test if the spatial means (and spatial variances) are equal across states. Within each state, we test if the spatial means (and spatial variances) are equal across censuses, and if the temporal means (and temporal variances) are equal across counties. In addition, for each type of TL, we test whether the spatial or temporal distributions of county population count are identical across states within each census, across censuses within each state, or across counties within each state, using the Anderson-Darling (AD) *k*-sample test (Scholz and Stephens 1987). We examine the independence of county population counts by testing the presence of spatial autocorrelation (across counties within each state) and temporal autocorrelation (across censuses within each county), using the global Moran's I (Cliff and Ord 1981) and Ljung-Box test (Ljung and Box 1978) respectively.

We implement the AD test using the ad.test function from the kSamples package in R (Scholz and Zhu 2019), with p-value estimated from 1,000 simulated AD statistics. We test the significance of the global Moran's I using the moransI function of the lctools package in R (Kalogirou 2019). We use the binary weighting matrix and set the number of neighbors to be eight, assuming the shape of each county is rectangular. Based on this spatial structure, within a unique combination of census and state, the number of counties with finite count values must be at least nine. 842/958 ≈ 88% combinations of censuses and states satisfy this condition. P-value of the Moran's I is obtained from the randomization null hypothesis test (Goodchild 1986). We test and show the effect of different weighting schemes and number of neighbors on Moran's I and its significance in Appendix S4. On the other hand, we test the temporal correlation of county population count time series for each county with at least five non-zero counts in all censuses, using lags from one to five separately. We carry out the test using the Box.test function in R (R Core Team 2019).

Within a census, spatial means of county population count differ significantly across states with one exception. Analysis of variance (anova) rejects the hypothesis that the spatial means are equal across states in all censuses, except 1790 (p-value = 0.4414) (Table S10). The Bartlett test rejects the hypothesis that spatial variances of county count are equal across states for each census (Table S13). The Anderson-Darling (AD) test rejects the hypothesis that the county population counts follow the same distribution across states for each census (Table S16).

Within a state, spatial means are significantly different across censuses by anova (p-value < 0.05) in 52 of the 55 states (Table S11). Spatial variances are significantly different across censuses by the Bartlett test in 52 of the 55 states (except Alaska Territory, Hawaii, and New Mexico Territory) (Table S14). The AD test rejects the null hypothesis that the county population count follows the same spatial distribution across censuses for 49 of the 55 states (except Alaska, Alaska Territory, Hawaii, Hawaii Territory, Maine, and Montana) (Table S17).

Within a state, temporal means differ significantly across counties by anova in 57 of the 64 states (except Alaska Territory, Dakota Territory, Idaho Territory, Michigan Territory, Oklahoma Territory, Washington Territory, and Wyoming Territory) (Table S12). Temporal variances differ significantly across counties by the Bartlett test in 57 of the 64 states (except Arkansas Territory, Dakota Territory, Florida Territory, Idaho Territory, Michigan Territory, Oklahoma Territory, and Wyoming Territory) (Table S15). The AD test rejects the null hypothesis that the historical county population count follows the same temporal distribution across counties for 58 of the 64 states (except Dakota Territory, Idaho Territory, Michigan Territory, Oklahoma Territory, Washington Territory, and Wyoming Territory) (Table S18).

Of the 842 unique combinations of censuses and states with at least nine counties, Moran's I is tested for significance in 835 combinations (seven combinations have undefined test statistics). Overall, 259 of the 835 census-state combinations have significant Moran's I (randomization p-value < 0.05) (Table S19). Within a census, the proportion of significant Moran's I ranges from 0.2 to 0.5. On the other hand, 3186 counties contain at least five censuses with non-zero count measurements and were tested for temporal autocorrelation (Table S20). 47 counties have five censuses and are eliminated from the test with lag of five. The number (percentage) of counties with significant temporal autocorrelation for lags from one to five is 2813 (≈88%), 2659 (≈83%), 2489 (≈78%), 2407 (≈76%), and 2369 (2369/(3186-47) ≈ 75%) respectively.

We use CX's eqn 3 to estimate TL's slope *b* and CX's eqn 5 to estimate the standard error of *b*, regardless of the validity of the iid assumption. For each type of TL, we treat the county population count in the census as the true population distribution, and calculate its skewness, kurtosis, and coefficient of variation. For the spatial hierarchical TL, in each census, we include all counties in the US. For the spatial TL and the temporal TL, in a given state, we use all counties in all censuses. Distributions for the last two types of TL differ because they are constrained by different minimum data requirements to reduce sampling variation: the spatial TL requires that the number of censuses in a given state be at least five, while the temporal TL requires that the number of counties in a given state be at least five.

### B. Tweedie's exponential dispersion models

Following the theoretical works of Tweedie (1946, 1947, 1984), Kendal and Jørgensen (2011a, 2011b) proposed several so-called Tweedie models as an explanation of TL. The Tweedie models are a collection of three-parameter probability distributions that obey TL with different values of the TL's slope *b*. Familiar distributions among the Tweedie models are the normal distribution (*b*=0, conditional on a changing location parameter [mean] and constant scale [variance] parameter), the Poisson distribution (*b*=1), the gamma distribution (*b*=2, conditional on a constant shape parameter and varying scale parameter), and the inverse gamma or Wald distribution (*b*=3, conditional on a constant shape parameter and varying scale parameter). The probability distributions in the family of Tweedie models that have the greatest empirical relevance are called the compound Poisson-gamma (PG) distributions. A PG distribution is defined as a sum of a Poisson-distributed number of iid gamma random variables. Kendal and Jørgensen assert that all PG distributions generate TL with 1 < *b* < 2 but do not state conditions that make this true (for this or the other distributions mentioned above). For 0 < *b* < 1, no Tweedie distribution exists.

We assess the goodness of fit of the Tweedie model with four competing probability distributions commonly used to study species abundance distributions in ecology (Baldridge et al. 2016): log-series, Poisson lognormal (a Poisson distribution in which the single parameter is lognormally distributed), negative binomial, and Zipf distributions. For each TL, we fit these five distributions (Tweedie and the four competitors) to each observed distribution of county population count using the maximum likelihood method, and select the best-fitting model with the smallest AICc. We fit the log-series, Poisson lognormal, negative binomial, and Zipf distribution models using the "sads" package in R (Prado et al. 2018). We describe below the software used to fit the Tweedie distribution. If the Tweedie model is not selected as the best one, then it suggests that the Tweedie distribution may not describe the observed distribution well. Consequently, TL's slope predicted from the Tweedie model may disagree with the value estimated from the OLS.

The Poisson lognormal model and the Tweedie model are superior to other models in describing the spatial and temporal distributions of county population count. Specifically, for a census with at least five distinct states, the AICc weights of the Poisson lognormal model and the Tweedie model are higher than 0.9 in respectively 16 and seven of the 23 censuses (Table S21). The average AICc weights for the five tested distribution models are 0 for the log-series, 0 for the negative binomial, 0.69 for the Poisson lognormal, 0.31 for the Tweedie, and 0 for the Zipf.

The analysis in the next two paragraphs tests the Tweedie model against county population count within a state constrained by two different conditions: first, at least five censuses; second, at least five counties. These constraints are those used when we fit the descriptive LS models for the spatial TL and the temporal TL respectively.

County population count is present in at least five distinct censuses for each of 56 states. The AICc weights of the Tweedie model and the Poisson lognormal model are higher than 0.9 in respectively 37 and 15 of the 56 states (Table S22). In the remaining four states, AICc weight of the Tweedie model is 0.36 (Alaska Territory), 0.88 (Arizona Territory), 0.79 (New Mexico), and 0.20 (South Carolina). AICc weight of the Poisson lognormal model is 0.64 (Alaska Territory), 0.01 (Arizona Territory), 0.21 (New Mexico), and 0.80 (South Carolina). The average AICc weights for the five tested distribution models are 0 for the log-series, 0 for the negative binomial, 0.29 for the Poisson lognormal, 0.71 for the Tweedie, and 0 for the Zipf.

County population count is present in at least five counties in 79 states. The AICc weights of the Poisson lognormal model and the Tweedie model and are higher than 0.9 in respectively 29 and 19 of the 79 states (Table S23). Of the other 31 states, AICc weight of the Poisson lognormal model ranges from 0 to 0.86. AICc weight of the Tweedie model ranges from 0.11 to 0.88. The average AICc weights for the five tested distribution models are 0 for the log-series, 0 for the negative binomial, 0.51 for the Poisson lognormal, 0.39 for the Tweedie, and 0.1 for the Zipf.

To predict TL's slope, we fit the Tweedie model to county population count distributions. For the spatial hierarchical TL, we fit the Tweedie model to the distribution of county population count of all counties in the US for a given census. For the spatial TL and the temporal TL, we fit the Tweedie model to the distribution of historical (of all censuses) county population count of all counties in a given state.

We fit the Tweedie model and obtain its AICc respectively using the tweedie.profile function and the AICtweedie function from the "tweedie" package in R (Dunn and Smyth 2005; Dunn and Smyth 2008; Dunn 2017). We use the default inversion method of the tweedie.profile function to find TL's *b* at which the likelihood function is maximized (Dunn and Smyth 2008). When fitting the model we delete counties with zero count, so that the tweedie.profile function can estimate any *b* greater than one. This is different from the compound PG distribution that can accommodate *b* in the range from one to two only (Jørgensen 1997). The deletion of counties with a zero count will overestimate the means and may underestimate the variances, leading to biased estimate of TL's *b*. We believe that such a potential effect is negligible since the number of counties with zero counted people (72) is small compared to the total number of county counts (52,868), representing 0.14% of the count data.

We approximate the standard error of *b* assuming that the sampling distribution of the likelihood function values are normal, using the likelihood function percentiles. Specifically, we first divide the paired data (interpolated *b* and corresponding likelihood value L) into two groups, called "left" and "right," by the point with the maximal likelihood value, denoted by (*b*_max_, L_max_). The left group consists of pairs (*b*, L) such that L < L_max_ and *b* < *b*_max_. The right group consists of paired data such that L < L_max_ and *b* > *b*_max_. Then we find the *b* at which the 5% percentile of likelihood is reached for the left group (denoted as *b*_lower_) and the *b* at which the 95% percentile of likelihood is reached for the right group (denoted as *b*_upper_). Next we calculate the standard error of *b* as (*b*_upper_ - *b*_lower_)/4, assuming that estimates of *b* are approximately normally distributed. We choose the range of the interpolated *b* according to their regression estimates. Specifically, for each type of TL, we calculate the 25th percentile (Q1) and 75th percentile (Q3) of the regression estimates of *b*, and set up the range of interpolated *b* from Q1-3×(Q3-Q1) to Q3+3×(Q3-Q1) to accommodate a wide range of values. For example, in the spatial hierarchical TL, the regression estimates of *b* are from 1.52 to 2.44, with Q1 = 1.78 and Q3 = 2.14. The interpolated *b* in the Tweedie model ranges from 0.71 (≈1.78–3×(2.14–1.78)) to 3.21 (≈2.14 + 3×(2.14–1.78)). Depending on the TL types, censuses, and states, the increment of interpolated *b* is between 0.0245 and 0.0939. Any interpolated *b* less than or equal to one is removed during fitting (since these values are theoretically impossible according to the Tweedie model). We do not test for the consistency in the derived interval estimates using different fitting methods or different ranges of interpolated *b* for the same type of TL.

### C. Feasible sets of integer partitions

Xiao, Locey, and White (Xiao et al. 2015, hereafter XLW) used restricted integer partitions from number theory to model TL. A feasible set is defined as the set of all possible partitions of a given integer into a given number of parts, either ordered or unordered. Here we consider only unordered parts (including zero parts). Treating the given integer as the total population count within a state in a census (or the total historical population count within a county of a state) and the given number of parts as the number of counties in that state of that census (or the number of censuses for that county), the partition model (hereafter the XLW model) can be used to test the three types of TL for the US county population count. The XLW model assumes that the observed county population distribution is a realization of the unordered list of nonnegative integers from the set of permitted partitions. The appearance of each unique partition is assumed to be equally likely.

For example, suppose there are two censuses (1 and 2) and each census include two states (A and B). In census 1, state A has 1 person and 2 counties and state B has 3 people and 2 counties. In census 2, state A has 2 people and 2 counties and state B has 4 people and 2 counties. In census 1 and state A, the partition of 1 person (integer) into 2 counties (parts) is (1, 0). In census 1 and state B, the partitions of 3 people (integer) into 2 counties (parts) are (3, 0) and (2, 1). In census 2 and state A, the partitions of 2 people (integer) into 2 counties (parts) are (2, 0) and (1, 1). In census 2 and state B, the partitions of 4 people (integer) into 2 counties (parts) are (4, 0), (3, 1), and (2, 2). (In this unrealistic illustrative example, we are ignoring all constraints of minimum sample size.) Using the first partition from each combination of census and state for this illustrative example, we calculate the spatial mean and spatial variance as 0.5 and 0.25 respectively for census 1 and state A, 1.5 and 2.25 for census 1 and state B, 1 and 1 for census 2 and state A, 2 and 4 for census 2 and state B. In census 1, a slope of the spatial hierarchical TL can be calculated from the mean-variance pairs as [log(2.25)-log(0.25)]/[log(1.5)-log(0.5)] = 2. In state A, a slope of the spatial TL can be calculated from the mean-variance pairs as [log(1)-log(0.25)]/[log(1)-log(0.5)] = 2. Similarly, a slope of the temporal TL can be calculated by partitioning the total historical county population count into multiple censuses. Detailed calculation of the temporal TL slope will not be shown.

We first check the XLW model assumption by comparing the sampling distribution of the size of parts in a partition with the observed county population distribution. Specifically, for each combination of state and census with at least five counties, we generate 1,000 random partitions for the corresponding state population count into the number of counties within that state. Similarly, for each county within a state with at least five censuses, we generate 1,000 random partitions for the total historical county population count into the number of censuses for that county. We then tally the size of all parts in each set of 1,000 partitions and treat this collection of part sizes as the sampling distribution for county population count based on the XLW model. We then compare the sampling distribution with the corresponding observed distribution to infer if they follow the same distribution using the two-sample Kolmogorov-Smirnov (KS) test. If the test result is significant (p-value < 0.05), then we reject the null hypothesis that the XLW model can reproduce the observed county population count distribution. Consequently, the predicted TL slope may deviate from the observed value.

Within 972 unique combinations of states and censuses, the predicted spatial distribution of county population count from the XLW model differs significantly from the observed spatial distribution (KS test p-value < 0.05) in 599 combinations (≈ 61.6%) (Table S24). Within a state, the proportion of censuses with a significant difference between the observed spatial distribution and the spatial distribution predicted from the XLW model is above 0.5 in 48 of the 81 states that are tested. Within a census, the proportion of states with significant difference between the observed spatial distribution and the spatial distribution predicted from the XLW model is above 0.5 in 20 of the 23 censuses (except 1790, 1800, and 1810).

For 4051 unique counties, the predicted temporal distribution of county population count from the XLW model differs significantly from the observed temporal distribution in 1947 combinations (≈ 48.1%) (Table S25). Within a state, the proportion of censuses with significant difference between the observed temporal distribution and the temporal distribution predicted from the XLW model is above 0.5 in 32 of the 81 states that are tested.

Because we use the observed state (or total historical) population count as the integer and the observed number of counties (or number of censuses) as the number of parts, the XLW model guarantees that each random partition results in a predicted mean that equals exactly the observed value. For each observed mean-variance pair, the above 1,000 random partitions yield 1,000 mean-variance pairs (with identical mean). These 1,000 mean-variance pairs can be used to predict 1,000 TL's slopes for each empirically tested TL. Due to the variation in sample size, we only examine each mean-variance relationship that corresponds to at least five observed finite log(mean)-log(variance) pairs (see *Descriptive models of Taylor's law*). For each empirical TL, this procedure will yield at most 1,000 simulated TL and therefore at most 1,000 TL's slopes predicted from the XLW model. We calculate the point estimate and standard error of *b* of the XLW model as the median and the standard deviation of these slope estimates respectively.

Since the state (or total historical county) population counts are in the millions, it would be time-consuming to generate exact random partitions. Instead, we round each state population count (or total historical county population count) to its nearest thousand people and apply the XLW model to the rounded data. Although rounding can result in errors in the simulated variances and may bias the predicted TL slopes, we believe that such effect is negligible since the resulting bias may cancel out through rounding. Another reason we choose this procedure is that TL's slope is a scale-invariant parameter: change of unit in the tested variable (i.e., from every person to every thousand people) will not change the empirical TL's slope, provided TL holds (Jørgensen 1997). We do not know, and did not establish, whether the predictions of the XLW model are, similarly, scale-invariant.

We now give a hypothetical example that illustrates a way to tackle this question. This example suggests that TL's slope is not invariant under rescaling of the total population size in the XLW model but that suggestion is by no means definitive. Suppose the population counts of a hypothetical state in five consecutive censuses are respectively 10000, 20000, 40000, 80000, and 160000, and the number of counties in the state is 50 for all five censuses. For each census, using 100 simulations of random partitions of the state population count into 50 county population counts, we find that that 95% percentile interval for the slope (with lower and upper bounds as 2.5% and 97.5% percentiles respectively) of the spatial TL is (1.76, 2.28). Repeating the procedure for state population counts divided by 1000 (i.e., 10, 20, 40, 80, and 160 in censuses 1-5 respectively), we find that the 95% percentile interval for the slope of the spatial TL is (0.97, 1.88). Although the 95% intervals overlap, the distribution of TL slope using the rescaled counts seems shifted to the left compared to the distribution of TL slope using the original counts.

We use the rand_partitions function from the "rpartitions" package in R (Locey and McGlinn 2012) to simulate the random partitions of the XLW model including zero parts.

# Appendix S3: Assumption checking and model comparison under Bonferroni's correction

### A. Assumption checking of the predictive models

Bonferroni corrections lead to slight changes in the proportion of significant difference of county population count distribution at various scales. On the other hand, Bonferroni corrections result in substantial decreases in the proportion of significant spatial or temporal autocorrelation of the county population count distribution.

When county population count distributions are compared across states within each of 23 censuses, the significance level of hypothesis testing changes to 0.05/23 (≈ 0.0022) according to the Bonferroni correction. Analysis of variance, Bartlett test, and Anderson-Darling test yield results that are the same as those without Bonferroni correction (Tables S10, S13, and S16).

When county population count distributions are compared across censuses within each of the 55 states, the significance level changes to 0.05/55 (≈ 0.00091) according to the Bonferroni correction. Compared to the results without Bonferroni correction, the difference in mean is not significant in Arizona Territory and Hawaii Territory by the analysis of variance, resulting in significant difference in mean in 50 states (Table S11). The Bartlett test does not reject the hypothesis of equal variance of county population count in Arizona Territory and in Hawaii Territory, leaving 50 states with significant difference in variance (Table S14). The Anderson-Darling test does not reject an identical county population count distribution across censuses in Arizona Territory, New Hampshire, New Mexico Territory, Utah, Utah Territory, and Vermont, leaving 43 states with significant differences in distribution (Table S17).

When historical county population count distributions are compared across counties within each of the 64 states, the significance level changes to 0.05/64 (≈ 0.00078) according to the Bonferroni correction. Compared to the results without Bonferroni correction, analysis of variance does not reject the equality of mean in Arizona Territory, Arkansas Territory, and Montana Territory, leaving 54 states showing significant differences in mean (Table S12). The Bartlett test does not reject the equality of variance in Alaska Territory, Arizona Territory, Montana Territory, New Mexico Territory, and Washington Territory, leaving 52 states showing significant differences in variance (Table S15). The Anderson-Darling test does not reject the hypothesis of identical distributions across counties in Alaska Territory, Arizona Territory, Montana Territory, and Utah Territory, leaving 54 states showing significant differences in distribution (Table S18).

Spatial autocorrelation of county population count is tested using Moran's I for each of 835 combinations of censuses and states, resulting in a corrected significance level of 0.05/835 (≈ 0.00005988). Compared to the result without Bonferroni correction, the proportion of combinations with significant spatial autocorrelation reduces from 259/835 (≈ 31%) to 108/835 (≈ 13%) (Table S19). Within each census, the significance level is adjusted to 0.05/# of states in that census, and the proportion of states with significant spatial autocorrelation ranges from 8.7% to 33%.

Temporal autocorrelation of historical county population count is tested using Box-Ljung test with lags of one through four for 3186 counties (with corrected significance level of 0.05/3186 ≈ 0.00001569), and with lag of five for 3139 counties (with corrected significance level of 0.05/3139 ≈0.00001593). Compared to the result without Bonferroni correction, the proportion of combinations with significant temporal autocorrelation reduces to 146/3186 (≈ 4.6%) for lag-1, 683/3186 (21%) for lag-2, 743/3186 (23%) for lag-3, 680/3186 (21%) for lag-4, and 594/3139 (19%) for lag-5 (Table S20).

Despite the drop in the proportion of significant spatial and temporal autocorrelation after Bonferroni corrections, these results do not disprove the existence of autocorrelation (since decreased Type I error (false positive) can inflate Type II error (false negative), making the significant autocorrelation less discoverable).

When constructing the XLW model to predict TL, we use the Kolmogorov-Smirnov test to compare whether the observed and the predicted county population count distributions are identical across various scales. When compared within each of the 972 combinations of censuses and states (including zero counts), the corrected significance level becomes 0.05/972 (≈ 0.00005144) and the proportion of combinations showing significant differences is 332/972 (≈ 34%) (Table S24). When compared within each of the 4051 counties (including zero counts), the corrected significance level becomes 0.05/4051 (≈ 0.00001234) and the proportion of combinations showing significant differences is 62/4051 (≈ 1.5%) (Table S25).

### B. Model prediction of TL's slope

For each combination of type of TL and model, we calculate the interval estimate of the slope using adjusted critical *t* value (= *t*_0.025/(# of tests), n-1_) using the Bonferroni correction. We use the adjusted interval estimate to quantify the predicted TL's slope values.

For the spatial hierarchical TL, after Bonferroni correction (# of tests = 23), the slope predicted by the CX model (*b*_CX_) is significantly higher than one in all censuses and significantly higher than two in all but two (1820 and 1830) censuses (Table S36). Slope predicted by the Tweedie model (*b*_T_) is not significantly different from one in ten censuses and not significantly different from two in all censuses. Slope predicted by the XLW model (*b*_XLW_) is significantly lower than two in 17 censuses and not significantly different from two in the other five censuses.

For the spatial TL, after Bonferroni correction (# of tests = 55), *b*_CX_ is significantly positive in all but seven states (Alaska, Alaska Territory, Arizona Territory, Hawaii, Hawaii Territory, New Mexico Territory, and Utah Territory) (Table S37). *b*_T_ is significantly positive in 14 states and not significantly different from zero in 41 states. *b*_XLW_ is significantly positive in all but nine states (Alaska, Alaska Territory, Arizona Territory, Hawaii, Hawaii Territory, New Mexico Territory, Montana, South Dakota, and Utah Territory).

For the temporal TL, after Bonferroni correction (# of tests = 64), *b*_CX_ is significantly positive in 49 states and not significantly different from zero in 15 states (Table S38). *b*_T_ is significantly positive in 38 states and not significantly different from zero in 26 states. *b*_XLW_ is significantly positive in all but nine states (Alaska Territory, Arizona Territory, Arkansas Territory, Florida Territory, Idaho Territory, Michigan Territory, Montana Territory, Oklahoma Territory, and Wyoming Territory).

Like the results from the assumption checking, the slope comparison after Bonferroni correction shows a smaller proportion of significant difference than that without Bonferroni correction. This observation does not imply the absence of significant difference due to the possibility of inflated Type-II error (false negative).

# Appendix S4: Moran's I under various weighting schemes and number of neighbors

We apply different weighting schemes and number of neighbors in the Moran's I test to examine their effects on the spatial autocorrelation of county population counts within each state for each census. Overall, we do not find substantial differences in the proportion of significant spatial autocorrelation using the different methods (Table S29).

We define the number of neighbors of a given county as eight and the weighting scheme to be binary (weight of 1 for the neighbors and weight of 0 otherwise) in the main text. Here we repeat the Moran's I test using four neighbors with binary weighting, eight neighbors with bi-square weighting (weight = (1-(distance of a neighbor/distance of the furthest neighbor)^2^)^2^ or 0 otherwise), four neighbors with bi-square weighting, and all counties in a state as neighbors with bi-square weighting.

Among all combinations of states and censuses examined, the proportion of significant Moran's I is (in descending order) 259/835 (≈ 31.0%) for eight neighbors with binary weighting, 281/910 (≈ 30.9%) for all counties as neighbors with bi-square weighting, 239/837 (≈ 28.6%) for eight neighbors with bi-square weighting, 226/876 (≈ 25.8%) for four neighbors with binary weighting, and 212/906 (≈ 23.4%) for four neighbors with bi-square weighting. On average, the proportion of significant Moran's I within each census differs by at most 7.7% (between eight neighbors with binary weighting (32.3%) and four neighbors with bi-square weighting (24.6%)).

# Appendix S5: OLS and CX model using bootstrap samples of county population counts

### A. Methodology

To further test the CX model and the effect of spatial or temporal autocorrelation on TL, we take bootstrap samples of the census data at two statistical resolutions for each type of TL. First, we bootstrap samples of county population count from the level that the means and variances are calculated. That is, for the spatial hierarchical TL and spatial TL, county population counts are bootstrapped from all counties in a given state and census. For the temporal TL, county population counts are bootstrapped from all censuses in a given state and county. Second, we bootstrap samples from the level across which the means and variances are fitted to test TL. That is, for the spatial hierarchical TL, county population counts are bootstrapped from all counties of the US in a given census and then assigned randomly into a fixed number of pseudo states. For the spatial TL and temporal TL, county population counts from all censuses are bootstrapped from all censuses in all counties of a given state, then randomly assigned into a fixed number of pseudo censuses or a fixed number of pseudo counties respectively. The two different resolutions at which bootstrap samples are generated reflect different levels at which the spatial or temporal autocorrelation has been altered.

We also test the effect of sample size on the CX model by replicating the bootstrap samples with different numbers of sampling units (*n* = 15, 30, 60, 120, where *n* denotes the number of pseudo counties or pseudo censuses used to calculate one mean-variance pair) and mean-variance pairs (*N* = 5, 10, 20, 40, 80, where *N* denotes the number of mean-variance pairs used to test TL). We choose the smallest values of *n* (15) and *N* (5) at the thresholds recommended by Taylor et al. (1988) and double them sequentially. When samples are bootstrapped from the level at which a mean and a variance are calculated, the number of mean-variance pairs (*N*) equals the number of states in a given census from the census data (for the spatial hierarchical TL), the number of censuses in a given state from the census data (for the spatial TL), or the number of counties in a given state from the census data (for the temporal TL).

For each combination of TL, bootstrapping resolution, *n*, and *N*, we calculate 500 ordinary least-squares regression estimates of TL's slope (*b*) using 500 bootstrap samples of the census data (denoted by *b*_reg_). We also predict *b* using the CX model (eqn 3 in Cohen and Xu 2015) and the same 500 bootstrap samples (denoted by *b*_CX_). Comparison of *b*_reg_ and *b*_CX_ is done at the maximal *n* and *N* available (*n* = 120 and *N* = 80), because CX's formulas hold asymptotically at large numbers of sampling units and mean-variance pairs (Cohen and Xu 2015). We calculate the interval estimate of *b* using the 2.5% percentile as the lower bound and the 97.5% percentile as the upper bound from the corresponding set of 500 *b*_reg_ or 500 *b*_CX_.

### B. Implications of bootstrap sampling

Results from the bootstrap sampling experiments further reveal the effects of spatial and temporal autocorrelation on TL.

First, for the spatial hierarchical TL, when bootstrapping from within a census, *b*_reg_ estimated from the bootstrap samples is not significantly different from three in all censuses; while *b*_reg_ estimated from the census data is significantly less than three in all but one census (1790) (Table S30). This means that when the spatial correlation of the data is diminished by bootstrapping, TL's *b* increases. This makes sense because the slope of spatial hierarchical TL can be interpreted as a measure of the extent to which the population aggregates in space, and lower spatial correlation would mean less similar population abundance in nearby spatial locations, causing greater increase in the spatial variance for a unit increase in the spatial mean. On the other hand, when bootstrapping from within a unique combination of census and state, the average lower bounds of the 95% confidence interval of *b*_reg_ estimated from the bootstrap samples and from the census data are respectively 1.88 and 1.59. The average upper bounds of the 95% confidence interval of *b*_reg_ estimated from the bootstrap samples and from the census data are respectively 2.10 and 2.41 (Tables S30). This observation reflects that the slope of the spatial hierarchical TL is not affected by the spatial structure within a state as much as by that across states, since the spatial autocorrelation of county population counts among states is preserved under the bootstrapping within a combination of census and state.

For the spatial TL, bootstrapping from within a state leads to different comparisons in *b*_reg_ between the samples and the census data among states. For example, in Arkansas and California, bootstrap samples yield higher *b*_reg_ than the census data. The opposite is true for Delaware and Rhode Island. In Florida and Wyoming, we cannot determine whether the bootstrap samples and the census data yield different *b*_reg_ (Table S31). In contrast, when bootstrapping from within a unique combination of state and census, the average lower bounds of the 95% confidence interval of *b*_reg_ are 2.49 for the bootstrap samples and 2.17 for the census data. The average upper bounds of the 95% confidence interval of *b*_reg_ are 2.94 for the bootstrap samples and 3.30 for the census data. These observations show that random sampling from counties while keeping the temporal structure among censuses will not alter the slope of spatial TL. They also suggest that the effect of temporal autocorrelation of county population count (diminished by bootstrapping from all counties across all censuses within a state) on the spatial TL is nonuniform and state-specific.

Bootstrapping samples for the temporal TL leads to the same conclusion as for the spatial TL, meaning that reducing the spatial autocorrelation among counties within a state, not reducing the temporal autocorrelation of historical population counts within a county, will alter the slope of temporal TL (Table S32). The effect of spatial autocorrelation among counties on the temporal TL is state-specific.

On the other hand, when comparing *b*_reg_ and *b*_CX_ using the same bootstrap samples, we find that, for all types of TL, bootstrap sampling at the coarser levels (within census for the spatial hierarchical TL and within state for the spatial and the temporal TL) leads to smaller discrepancies between *b*_CX_ and *b*_reg_ than bootstrap sampling at the finer levels (within census-state combination for the spatial hierarchical and the spatial TL, and within state-county combination for the temporal TL) (Tables S30-S32). Specifically, for the spatial hierarchical TL, when bootstrapping within each combination of census and state, *b*_CX_ is greater than *b*_reg_ in all but two (1960 and 1970) of the 23 censuses; when bootstrapping within each census, *b*_CX_ is significantly different from *b*_reg_ in four censuses only (*b*_CX_ > *b*_reg_ in 1850, 1860, and 1970; *b*_CX_ < *b*_reg_ in 1790) (Table S30). For the spatial TL, when bootstrapping within each combination of census and state, *b*_CX_ differs from *b*_reg_ in 31 of the 55 states (*b*_CX_ > *b*_reg_ in 17 states and *b*_CX_ < *b*_reg_ in 14 states); when bootstrapping within each state, *b*_CX_ is smaller than *b*_reg_ in nine states (Table S31). For the temporal TL, when bootstrapping within each county, *b*_CX_ differs from *b*_reg_ in 57 of the 64 states (*b*_CX_ > *b*_reg_ in 31 states and *b*_CX_ < *b*_reg_ in 26 states); when bootstrapping within each state, *b*_CX_ is smaller than *b*_reg_ in nine states (Table S32).

This observation can be explained by the fact that bootstrapping at the finer levels preserves most of the spatial or temporal autocorrelations in the census data compared to bootstrapping at the coarser levels, leading to more severe violations of the assumptions of the CX model (iid) and greater discrepancies between *b*_reg_ and *b*_CX_ at the finer levels.

# Appendix S6: Descriptive least-squares models of TL with at least 15 units for each mean-variance pair

### A. Spatial hierarchical TL

Setting a lower bound of at least 15 counties to generate a mean-variance pair reduces the number of states within a census (for example, to 7 states in 1790 and to 44 states in 2010). The AICc of spatially correlated GLS linear regressions are smaller (i.e., the models are better fitting) than those of the corresponding OLS linear regressions (eqn 1) in the six most recent censuses (Table S33), whereas in the early censuses the OLS model is better according to the AICc. Specifically, the best linear model (with the least AICc) is the OLS regression in 17 censuses (1790-1950), GLS regression with rational quadratics spatial correlation in five censuses (1960, 1980-2010), and GLS regression with exponential spatial correlation in one census (1970). In all censuses the slope of the best linear model is significantly positive. The best GLS regressions yield slopes that are higher (but not significantly higher) than the slope estimated from the corresponding OLS regressions. Compared to the best linear model without the constraint of at least 15 units for each mean-variance pair, the slope estimate of the best linear model with the constraint is lower (but may not be significantly lower) and higher (but may not be significantly higher) in four and 19 censuses respectively. The magnitude of the average difference in estimated slope across all 23 censuses is 0.1845.

### B. Spatial TL

The constraint of at least 15 counties to generate a mean-variance pair sometimes reduces the number of censuses within a state (e.g., to 6 censuses in Alaska and not at all in six states, which still have 23 censuses). We fit the linear regression models in 42 states separately, each with at least five censuses. The AICc of temporally correlated GLS linear regressions are smaller than those of the corresponding OLS linear regressions in the 38 of the 42 states (Table S34). Specifically, the best linear model selected by AICc is the GLS regression with a first-order autoregressive process in 23 states, GLS regression with a first-order moving average process in one state, and GLS regression with a first-order autoregressive moving average process in 14 states. The best GLS models yield significantly positive spatial TL slopes in all states except South Dakota. The best linear models without constraint and with constraint are obtained in 31 states. The magnitude of the average difference in slope across the 31 states is 0.0965.

### C. Temporal TL

The constraint of at least 15 censuses to generate a mean-variance pair reduces the number of counties within a state (to 5 counties in Rhode Island and to 141 counties in Texas). We fit the linear regression models in 36 states separately, each with at least five counties. The AICc of spatially correlated GLS linear regressions are smaller than those of the corresponding OLS linear regressions in 20 of the 36 states (Table S35). Specifically, the best linear model is OLS regression in 16 states, GLS regression with exponential spatial correlation in 13 states, GLS regression with Gaussian spatial correlation in two states, and GLS regression with rational quadratics spatial correlation in five states. In all 36 states the best linear model yields significantly positive TL slope. Compared to the best linear models without the constraint of at least 15 censuses, the slope estimate with the constraint is smaller (but may not be significantly) and greater (but may not be significantly) in 14 and 13 states respectively. The magnitude of the average difference in slopes across all 36 states is 0.011.

# References

Baldridge E, Harris DJ, Xiao X, White EP. An extensive comparison of species-abundance distribution models. PeerJ 2016; 4: e2823.

Cliff AD, Ord JK. (1981). Spatial processes: models and applications. London: Pion; 1981.

Cohen JE, Xu M. Random sampling of skewed distributions implies Taylor’s power law of fluctuation scaling. Proc Natl Acad Sci USA. 2015; 112: 7749–7754.

Dunn PK, Smyth GK. Series evaluation of Tweedie exponential dispersion models. Stat Comput. 2005; 15: 267–280.

Dunn PK, Smyth GK. Evaluation of Tweedie exponential dispersion models using Fourier inversion. Stat Comput. 2008; 18: 73–86.

Dunn PK. Tweedie: evaluation of Tweedie exponential family models. R package version 2.3. 2007. Available from: https://CRAN.R-project.org/package=lctools

Goodchild MF. Spatial autocorrelation (Vol. 47). Geo Books; 1981.

Jørgensen B. The theory of dispersion models. London: CRC Press; 1997.

Kalogirou S. lctools: Local Correlation, Spatial Inequalities, Geographically Weighted Regression and Other Tools. R package version 0.2-7; 2019. Available from: [https://CRAN.R-project.org/package=lctools](https://cran.r-project.org/package=lctools)

Kendal WS, Jørgensen B. Taylor's power law and fluctuation scaling explained by a central-limit-like convergence. Phys Rev E. 2011a; 83: 066115.

Kendal WS, Jørgensen B. Tweedie convergence: A mathematical basis for Taylor's power law, 1/f noise, and multifractality. Phys Rev E. 2011b; 84: 066120.

Ljung GM, Box GE. On a measure of lack of fit in time series models. *Biometrika* 1978; 65: 297–303.

Locey K, McGlinn D. rpartitions: code for integer partitioning. Weecology, Logan, UT. R package version 0.1. 2012. Available from: <https://github.com/klocey/partitions>.

Prado PI, Miranda MD, Chalom A. sads: Maximum Likelihood Models for Species Abundance Distributions. R package version 0.4.2. 2018. Available from: https://CRAN.R-project.org/package=sads

R Core Team. R: A language and environment for statistical computing. R Foundation for Statistical Computing, Vienna, Austria. 2019. Available from: https://www.R-project.org/.

Reuman DC, Zhao L, Sheppard LW, Reid PC, Cohen JE. Synchrony affects Taylor’s law in theory and data. Proc Natl Acad Sci USA. 2017; 114: 6788–6793.

Scholz FW, Stephens MA. K-sample Anderson–Darling tests. J Am Stat Assoc. 1987; 82: 918–924.

Scholz F, Zhu A. kSamples: K-Sample Rank Tests and their Combinations. R package version 1.2-9. 2019. Available from: [https://CRAN.R-project.org/package=kSamples](https://cran.r-project.org/package=kSamples)

Taylor LR, Perry JN, Woiwod IP, Taylor RAJ. Specificity of the spatial power-law exponent in ecology and agriculture. Nature. 1988; 332: 721–722.

Tweedie MCK. The regression of the sample variance on the sample mean. J London Math Soc. 1946; 1: 22–28.

Tweedie MCK. Functions of a statistical variate with given means, with special reference to Laplacian distributions. In: Mathematical Proceedings of the Cambridge Philosophical Society. Cambridge: Cambridge University Press; 1947. pp. 41–49.

Tweedie MCK. (1984). An index which distinguishes between some important exponential families. Proceedings of the Indian Statistical Institute Golden Jubilee International Conference on Statistics: Applications and New Directions 1984; 579: 579–604.

Xiao X, Locey KJ, White EP. A process-independent explanation for the general form of Taylor’s law. Am Nat. 2015; 186: E51–E60.

# Supplementary Table Captions

Table S1. Descriptive model summary of the spatial hierarchical TL for each census (year). n is the number of finite log(mean)-log(variance) pairs (here, the number of states) in each fitting. OLS is the ordinary least-squares linear regression and best LS is the least-squares linear regression with the smallest AICc. mod shows the name of the best LS. gls_exp and gls_rat are respectively the generalized least-squares linear regression with exponential spatial correlation and rational quadratics spatial correlation. est and std err are respectively the point estimate and standard error of the estimate. lower and upper are respectively the lower and upper bounds of the slope estimates with 95% confidence level. AICc and AICc wt are respectively the Akaike Information Criterion corrected for the number of parameters and the AICc weight of the corresponding model among all fitted models in a census. A model with the smaller AICc (or greater AICc wt) is better. cor is the correlation coefficient of the observed variance and the predicted variance from each model. ∆AICc is AICc of the best LS minus the AICc of the OLS.

Table S2. Correlation coefficient (r) between the observed variance and predicted variance using the least-squares regressions, for the spatial hierarchical TL tested in each census (year). mod lists gls_exp, gls_gauss, gls_lin, gls_rat, and gls_sph, which are respectively the generalized least-squares regressions with exponential, Gaussian, linear, rational quadratics, and spherical spatial correlation. ols is the ordinary least-squares regression.

Table S3. Interval estimate of the slope difference of spatial hierarchical TL between the best linear model (*b*_reg_) and the ordinary least-squares regression (*b*_ols_), Cohen-Xu model (*b*_CX_), Tweedie model (*b*_T_), and Xiao-Locey-White model (*b*_XLW_) in each census (Year). Each interval estimate is derived from 100 estimated slope differences using 100 bootstrap samples of the county population data. Lower and upper values are respectively the 2.5% and 97.5% percentiles of the 100 slope differences.

Table S4. Descriptive model summary of the spatial TL for each state. gls_ar1, gls_ma1, and gls_arma11 in the best LS are respectively the generalized least-squares linear regressions with first-order autoregressive component, first-order moving average component, and first-order autoregressive and first-order moving average components. n is the number of finite log(mean)-log(variance) pairs (here, the number of censuses) in each fitting. Other notations are defined as in Table S1.

Table S5. Correlation coefficient (r) between the observed variance and predicted variance using the least-squares regressions, for the spatial TL tested in each state. gls_ar1, gls_ma1, and gls_arma11 are respectively the generalized least-squares regressions with first-order autoregressive component, first-order moving-average component, and first-order autoregression and first-order moving-average component. ols and r are defined in Table S2.

Table S6. Interval estimate of the slope difference of spatial TL between the best linear model (*b*_reg_) and the ordinary least-squares regression (*b*_ols_), Cohen-Xu model (*b*_CX_), Tweedie model (*b*_T_), and Xiao-Locey-White model (*b*_XLW_) in each state (State). Table S3 gives the method for interval estimation.

Table S7. Descriptive model summary of the temporal TL for each state. gls_gauss, gls_sph, and gls_lin are respectively the generalized least-squares with Gaussian spatial correlation, spherical spatial correlation, and linear spatial correlation. n is the number of finite log(mean)-log(variance) pairs (here, the number of counties) in each fitting. Other notations are defined as in Table S1.

Table S8. Correlation coefficient (r) between the observed variance and predicted variance using the least-squares regressions, for the temporal TL tested in each state. mod and r are defined in Table S2.

Table S9. Interval estimate of the slope difference of temporal TL between the best linear model (*b*_reg_) and the ordinary least-squares regression (*b*_ols_), Cohen-Xu model (*b*_CX_), Tweedie model (*b*_T_), and Xiao-Locey-White model (*b*_XLW_) in each state (State). Table S3 gives the method for interval estimation.

Table S10. Results of one-way analysis of variance of the state-specific spatial means of county population counts among states within each census (year). df is the degrees of freedom. sumsq is the sum of squares, meansq is the mean sum of squares, statistic is the F statistic, and p_value is the p value for the corresponding test.

Table S11. Results of one-way analysis of variance of the spatial means of county population counts across censuses within each state. p_value is defined in Table S10.

Table S12. Results of one-way analysis of variance of the temporal means of county population counts across counties within each state. p_value is defined in Table S10.

Table S13. Bartlett test of homogeneity of the spatial variances of county population counts across states within each census (year). statistic is the chi-square test statistic. p_value is defined in Table S10.

Table S14. Bartlett test of homogeneity of the spatial variances of county population counts across censuses within each state. p_value is defined in Table S10.

Table S15. Bartlett test of homogeneity of the temporal variances of county population counts across counties within each state. p_value is defined in Table S10.

Table S16. Anderson-Darling test of identical spatial distribution of county population counts across states within each census. p_value is defined in Table S10.

Table S17. Anderson-Darling test of identical spatial distribution of county population counts across censuses within each state. p_value is defined in Table S10.

Table S18. Anderson-Darling test of identical temporal distribution of county population counts across counties within each state. p_value is defined in Table S10.

Table S19. Summary statistics of global Moran's I for spatial autocorrelation of county population counts within each combination of state and year. moran_exp and moran_obs are respectively the expected Moran's I and the observed Moran's I. p_ran and z_ran are respectively the p-value and the z score calculated from the randomization null hypothesis test (with undefined values denoted by NA). num_county is the number of counties.

Table S20. Summary statistics of the Ljung-Box test of temporal autocorrelation (lag 1 to lag 5) of historical county population counts over years, for each combination of state and county. num_census is the number of censuses. tcor_lagX (X=1,2,3,4,5) is the p-value of the test with a lag of 10X years.

Table S21. Summary statistics of five distribution models of county population counts within each census (year). size is the number of counties. lik_max is the approximate maximum likelihood of the Tweedie model. aicc_xxx gives the AICc of the Tweedie model (xxx=tweedie) and of each of the other four distributions models: log series distribution (xxx=logseries), negative binomial distribution (xxx=negbinom), Poisson-lognormal distribution (xxx=poilog), and Zipf distribution (xxx=zipf). wt_xxx gives the AICc weight of each of the five distribution models.

Table S22. Summary statistics of five distribution models of county population counts within each state (within at least five censuses in each state). size is the number of combinations of censuses and counties. Other columns are defined in Table S21.

Table S23. Summary statistics of five distribution models of county population counts within each state (within at least five counties in each state). size is the number of combinations of censuses and counties. Other columns are defined in Table S21.

Table S24. p-value of the 2-sample Kolmogorov-Smirnov (KS) test of the null hypothesis that the observed county population count distribution is identical to the predicted county population county distribution from the XLW model (including zero), for each combination of state and year. ks_p is the p-value of the KS test.

Table S25. p-value of the 2-sample Kolmogorov-Smirnov (KS) test of the null hypothesis that the observed historical county population count distribution is identical to the predicted historical county population county distribution from the XLW model (including zero), for every county. ks_p is the p-value of the KS test.

Table S26. Descriptive model summary of the spatial hierarchical quadratic TL for each census (year). OLS is the ordinary least-squares quadratic regression and best LS is the least-squares quadratic regression with the smallest AICc. n is the number of finite log(mean)-log(variance) pairs (here, the number of states) in each fitting. Other notations are defined in Table S1.

Table S27. Descriptive model summary of the spatial quadratic TL for each state. OLS is the ordinary least-squares quadratic regression and best LS is the least-squares quadratic regression with the smallest AICc. n is the number of finite log(mean)-log(variance) pairs (here, the number of censuses) in each fitting. Other notations are defined in Tables S1 and S4.

Table S28. Descriptive model summary of the temporal quadratic TL for each state. OLS is the ordinary least-squares quadratic regression and best LS is the least-squares quadratic regression with the smallest AICc. n is the number of finite log(mean)-log(variance) pairs (here, the number of counties) in each fitting. Other notations are defined in Tables S1 and S7.

Table S29. Proportion of states that show significant spatial autocorrelation of county population counts within each census, using five different ways of computing the Moran's I. Xneighbor_Y (X = 4 or 8, Y=binary or bisquare) is the binary (weight = 1 for distances less than or equal to the distance of the furthest neighbour, 0 otherwise) or bi-square (weight = (1-(distance of neighbor/distance of the furthest neighbor)^2)^2 or 0 otherwise) weighting scheme with 4 neighbors or 8 neighbors. allcounty_bisquare uses the bi-square weighting scheme for all counties in a state.

Table S30. Interval estimates of the spatial hierarchical TL slope (b) using bootstrap samples for each census (year), using the CX model (cx) and the ordinary least-squares (ols) regression separately. b and b_se are respectively the point estimate and the standard error of b obtained from the census data. b_boot_withinyear_lower (or upper) gives respectively the 95% lower (or upper) bound of b estimated from 500 samples bootstrapped within each year. b_boot_withinyearstate_lower (or upper) gives respectively the 95% lower (or upper) bound of b estimated from 500 samples bootstrapped within each combination of year and state.

Table S31. Interval estimates of the spatial TL slope (b) using bootstrap samples for each state, using the CX model (cx) and the ordinary least-squares (ols) regression separately. b and b_se are defined in Table S30. b_boot_withinstate_lower (or upper) gives respectively the 95% lower (or upper) bound of b estimated from 500 samples bootstrapped within each state. b_boot_withinstateyear_lower (or upper) gives respectively the 95% lower (or upper) bound of b estimated from 500 samples bootstrapped within each combination of state and year.

Table S32. Interval estimates of the temporal TL slope (b) using bootstrap samples for each state, using the CX model (cx) and the ordinary least-squares (ols) regression separately. b and b_se are defined in Table S30. b_boot_withinstate_lower (or upper) gives respectively the 95% lower (or upper) bound of b estimated from 500 samples bootstrapped within each state. b_boot_withinstatecounty_lower (or upper) gives respectively the 95% lower (or upper) bound of b estimated from 500 samples bootstrapped within each combination of state and county.

Table S33. Summary statistics of the best regression models (mod_best) of the spatial hierarchical TL for each census (year). n is the number of finite log(mean)-log(variance) pairs (here, the number of states) in each fitting. Each mean-variance pair is generated using at least 15 county population counts within a state. Model names are defined in Table S2. "est_slp" and "err_slp" are respectively the slope estimate and slope standard error. "aicc" is the model AICc.

Table S34. Summary statistics of the best regression models (mod_best) of the spatial TL for each state. n is the number of finite log(mean)-log(variance) pairs (here, the number of censuses) in each fitting. Each mean-variance pair is generated using at least 15 county population counts within a state. Model names are defined in Table S4. "est_slp", "err_slp" and "aicc" are defined in Table S33.

Table S35. Summary statistics of the best regression models (mod_best) of the temporal TL for each state. n is the number of finite log(mean)-log(variance) pairs (here, the number of counties) in each fitting. Each mean-variance pair is generated using at least 15 historical population counts of a county. Model names are defined in Table S2. "est_slp", "err_slp" and "aicc" are defined in Table S33.

Table S36. Spatial hierarchical TL's slope across five models in each census (year). The models are cx (Cohen-Xu model), reg_ols (ordinary least-squares linear regression), reg_best (least-squares linear regression with the smallest AICc), tweedie (Tweedie model), and xlw (Xiao-Locey-White model). n is the number of finite mean-variance pairs used in each model fitting. The last four columns show TL slope (b), standard error of the slope (b_se), and lower bound of the slope (b_lower) and upper bound of the slope (b_upper) using adjusted critical *t* value (upper tail probability is 0.025/23) according to the Bonferroni correction.

Table S37. Spatial TL's slope across five models in each state. Notations are defined as in Table S36. Lower and upper bounds of the slope are derived using adjusted critical *t* (upper tail probability is 0.025/55).

Table S38. Temporal TL's slope across five models in each state. Notations are defined as in Table S36. Lower and upper bounds of the slope are derived using adjusted critical *t* (upper tail probability is 0.025/64).

# Supplementary Figures and Figure Legends

### Figure S1


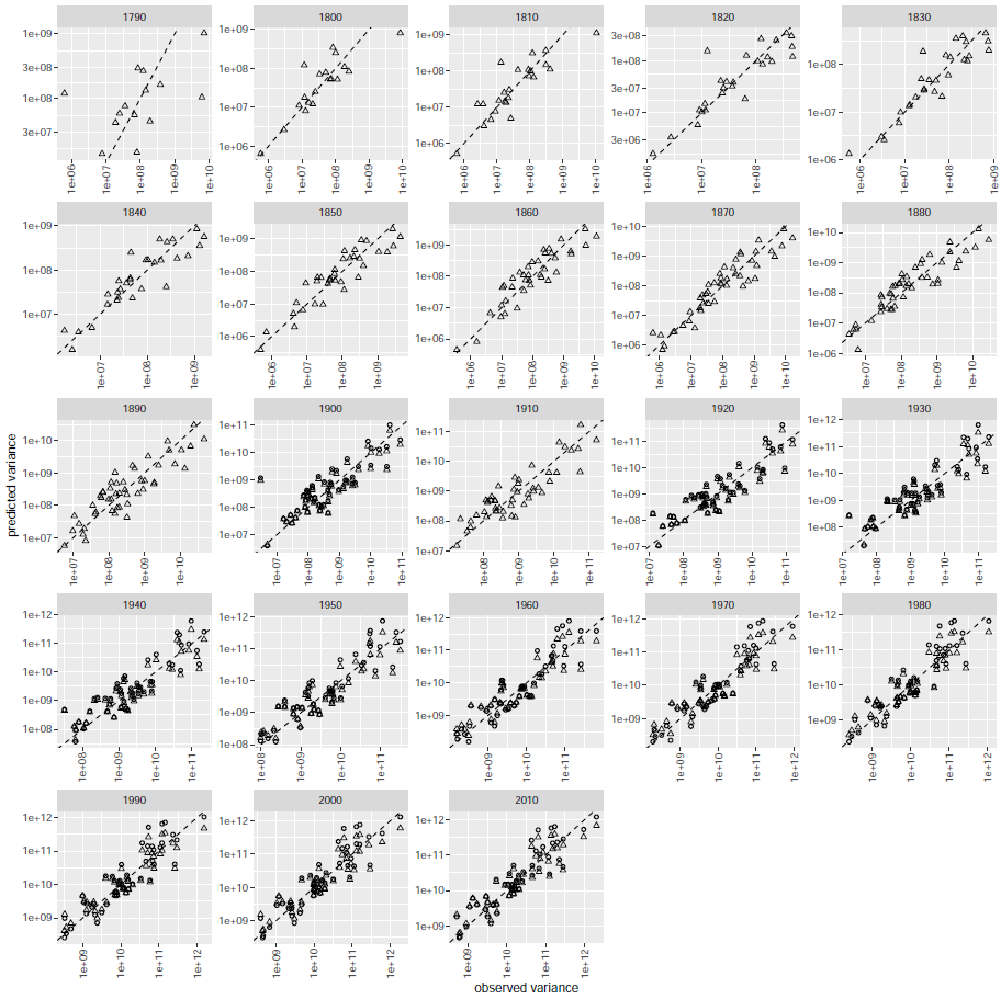


Figure S1. Predicted spatial variance of county population count against observed spatial variance for each census. Each marker shows one observed variance-predicted variance pair within a state in one census. Different markers denote the variance predicted from the ordinary least-squares regression (Δ) or the best least-squares regression with the smallest AICc (○). Predictions from the best least-squares regression are missing in some censuses because the ordinary least-squares is the best model. Dashed lines are the one-to-one reference lines.

### Figure S2


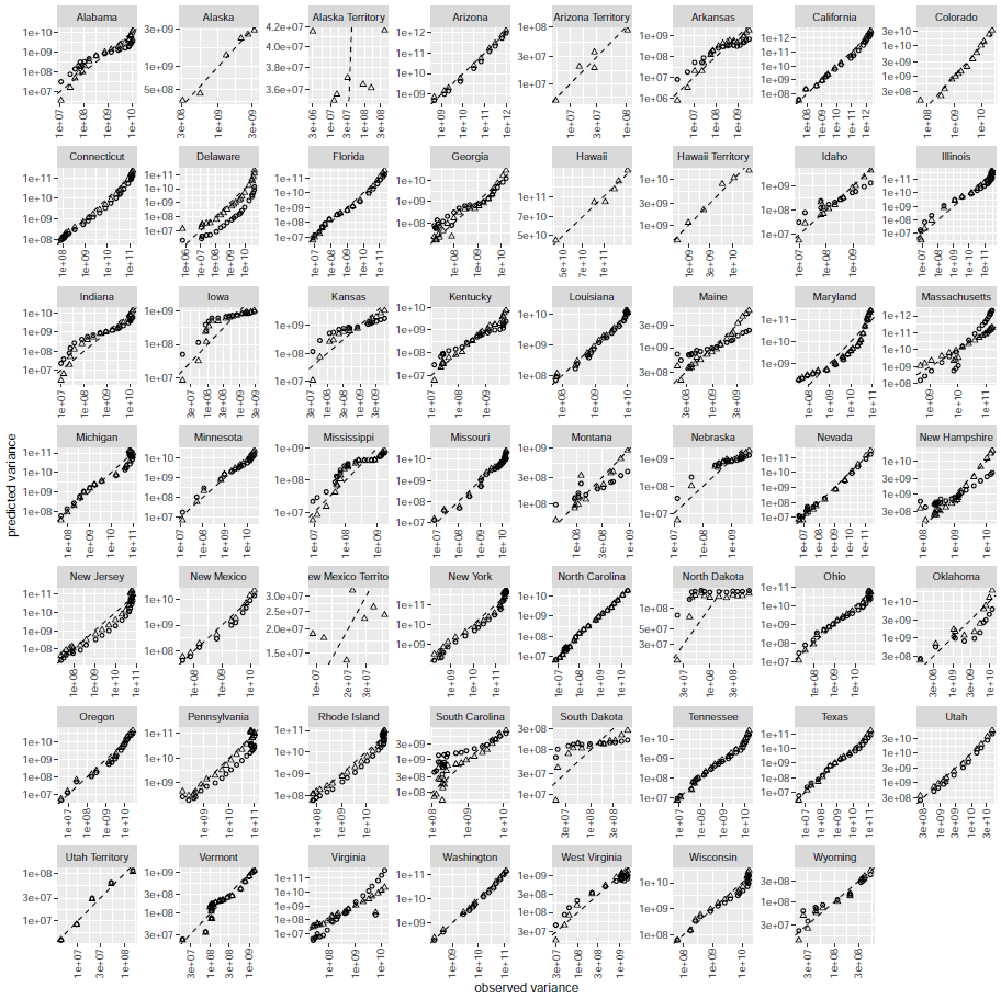


Figure S2. Predicted spatial variance of county population count against observed spatial variance for each state. Each marker shows one observed variance-predicted variance pair in one census within a state. Markers and lines are defined in Fig. S1. Predictions from the best least-squares regression are missing in some censuses because either the ordinary least-squares is the best model or the generalized least-squares regression fails to yield any prediction.

### Figure S3


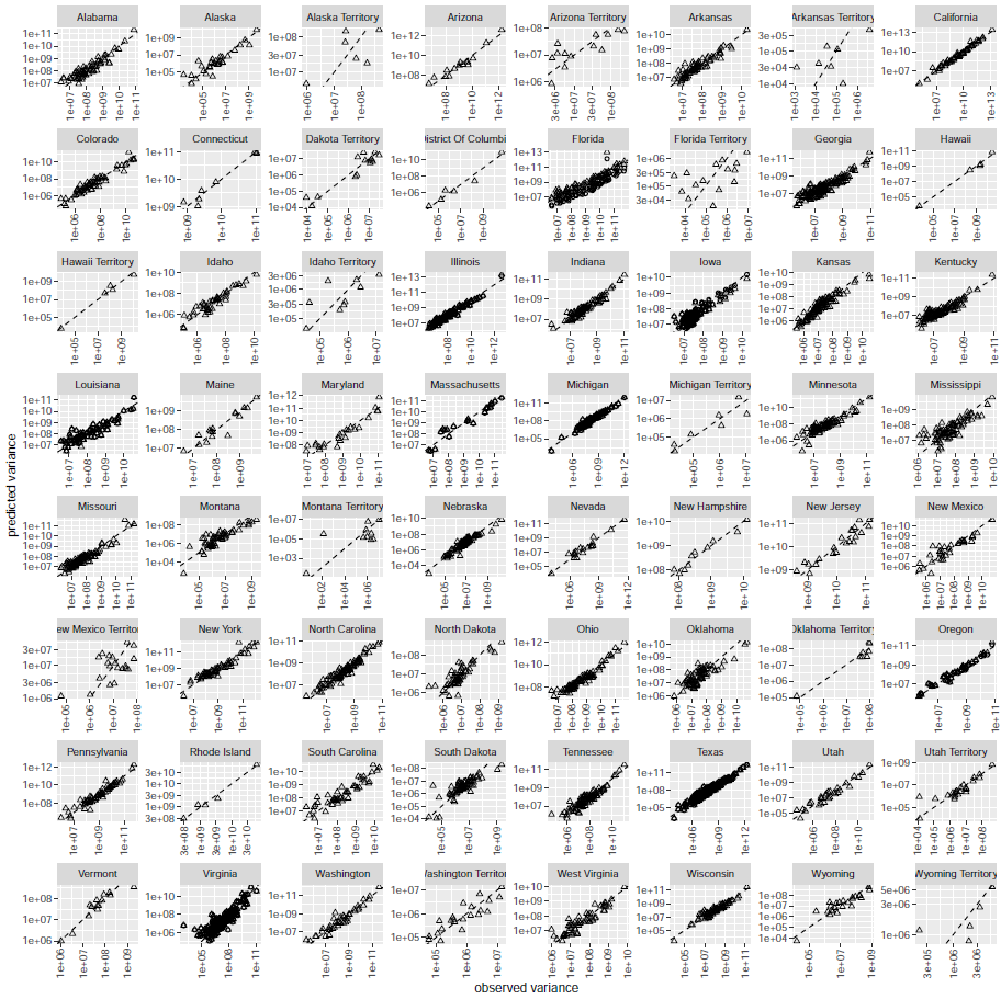


Figure S3. Predicted temporal variance of county population count against observed temporal variance for each state. Each marker shows one observed variance-predicted variance pair in one county within a state. Markers and lines are defined in Fig. S1. Predictions from the best least-squares regression are missing in some censuses because either the ordinary least-squares is the best model or the generalized least-squares regression fails to yield any prediction.
